# *Enigmacursor mollyborthwickae*, a neornithischian dinosaur from the Upper Jurassic Morrison Formation of the western USA

**DOI:** 10.1098/rsos.242195

**Published:** 2025-06-25

**Authors:** Susannah C. R. Maidment, Paul M. Barrett

**Affiliations:** ^1^ Fossil Reptiles, Amphibians and Birds Section, Natural History Museum, London, UK; ^2^ School of Geography, Earth and Environmental Sciences, University of Birmingham, Birmingham, UK; ^3^ Evolutionary Studies Institute, University of the Witwatersrand Johannesburg, Johannesburg, GP, South Africa; ^4^ Department of Earth Sciences, University College London, London, UK

**Keywords:** neornithischian, dinosaurs, Upper Jurassic, Morrison Formation, western USA, *Enigmacursor mollyborthwickae*

## Abstract

Although their remains have been known since the 1870s, the small, bipedal ornithischian dinosaurs from the Upper Jurassic Morrison Formation of the western USA remain poorly known. The historic type specimens are incomplete and poorly preserved and have recently been designated as *nomina dubia*. Here, we describe a recently collected, partial but three-dimensionally preserved skeleton of a new small-bodied ornithischian from the Morrison Formation of Colorado, USA, that we name *Enigmacursor mollyborthwickae* gen. et sp. nov. The skeleton includes substantial portions of the axial and appendicular skeleton and, when scored into a phylogenetic analysis, is shown to be a non-cerapodan neornithischian, whose closest relative is *Yandusaurus hongheensis* from the Late Jurassic of China. The discovery of *Enigmacursor* enhances the diversity of ornithischian dinosaurs from the Morrison Formation and provides new information on their anatomy. In addition, it demonstrates that there is additional cryptic diversity of small-bodied Morrison Formation ornithischians, suggesting they were a more diverse component of these Late Jurassic ecosystems than was previously realized.

## 1. Introduction

The Morrison Formation of western North America (Upper Jurassic: Kimmeridgian–Tithonian) has yielded a diverse, globally important fauna of non-avian dinosaurs (e.g. [1,2]). Although many Morrison Formation taxa are well known and have been intensively studied, some remain relatively obscure and have not been subjected to detailed scrutiny. Perhaps the most neglected group is composed of the small-bodied, bipedal ornithischian dinosaurs (see reviews in [3,4]). First described, in brief, by O. C. Marsh in the 1870s, all these taxa are based either on highly incomplete, fragmentary-type specimens (‘*Nanosaurus agilis’*, ‘*N*. (= *Othnielia*) *rex*’, ‘*Laosaurus* c*eler*’, ‘*L. gracilis'*, ‘*L*. (= *Othnielosaurus*) *consors’* [5–9]) or on material that is currently inaccessible for study (*Drinker nisti* [10]). Although previous workers have attempted to salvage these names by placing them in various combinations of synonymies, the proposal of new names and through referring other specimens to them (e.g. [4,11–15]), we recently demonstrated that none of the holotypes is adequate and that the referral of other specimens to these taxa is untenable [3]. Instead, these species should all be regarded as *nomina dubia* and set aside in the interest of taxonomic stability [3].

However, although the type specimens of Morrison small ornithischians are problematic, some of the other material collected from the formation has more promise for understanding the anatomy, taxonomy and diversity of these taxa. These include associated or articulated skeletons that consist of substantial portions of the postcranium and, rarely, cranial remains. The first to be mentioned was an articulated postcranial skeleton lacking skull from Emery County, Utah, which was described in brief by Galton & Jensen [14] and attributed tentatively to *Nanosaurus rex*. Since then several other significant specimens have come to light: a specimen from Howe Ranch, Wyoming nicknamed ‘Barbara’ (NMZ 1000010, formerly SMA 0010), which—despite its high completeness, including some skull material—remains undescribed, as it was formerly in a private collection [4], DMNH 21716 from Garden Park, Colorado, which was referred tentatively to *Othnielia rex* [11], but is problematic as it consisted of poorly preserved bone that was removed to leave natural moulds, and UW 24823, another skull-less postcranium from near Alcova, Wyoming [4]. None of these has received more than a short description (in some cases no description), so their contribution to understanding Morrison Formation species richness and ornithischian evolutionary history has been limited. This is unfortunate given the global scarcity of Middle–Late Jurassic non-thyreophoran ornithischians (e.g. [16]).

Here, we describe a recently collected specimen (NHMUK PV R 39000) from Moffat County, Colorado, which consists of a three-dimensionally preserved postcranial skeleton with some associated teeth. The aim of this article is to describe NHMUK PV R 39000, to assess its relationships with other ornithischians and to determine its implications for understanding the species richness of Morrison Formation ornithischian dinosaurs.

### 1.1. Institutional abbreviations

BYU, Brigham Young University, Provo, Utah, USA; CM, Carnegie Museum of Natural History, Pittsburgh, Pennsylvania, USA; DMNH, Denver Museum of Nature and Science, Colorado, USA; MWC, Dinosaur Journey Museum, Museums of Western Colorado, Fruita, USA; NHMUK, Natural History Museum, London, UK; NMZ, Natural History Museum of Zurich, Switzerland; SMA, Sauriermuseum, Aathal, Switzerland; USNM, National Museum of Natural History, Smithsonian Institution, Washington, DC, USA; UW, Geological Museum, University of Wyoming Laramie, Wyoming, USA; YPM, Peabody Museum, Yale University, New Haven, Connecticut, USA.

## 2. Methods

### 2.1. Phylogenetic methods

To determine the phylogenetic position of *Enigmacursor mollyborthwickae*, it was scored into the phylogenetic matrix of Han *et al*. [17]. This matrix was chosen because it was designed to establish the phylogenetic position of early diverging neornithischian dinosaurs, has a balanced sample of early diverging neornithischians, cerapodans, ornithopods and marginocephalians and the characters are clearly explained, documented and well illustrated. Two other major phylogenetic analyses of basal ornithischians have been published recently: Dieudonné *et al*. [18] and Fonseca *et al*. [19]. We chose not to use Dieudonné *et al*. [18] because their character list is problematic: many characters are difficult to operationalize due to insufficient documentation, and there are numerous examples of characters that score the same variation, including direct duplication of characters. In addition, this analysis is heavily focused on Ornithopoda, and lacks a balanced taxon sample of early diverging marginocephalians. The recent analysis of Fonseca *et al*. [19] was avoided because it is focused on basal dinosaur and basal ornithischian taxa and therefore includes numerous characters and taxa irrelevant to determining the phylogenetic position of *Enigmacursor*.

The data matrix used herein was assembled in Mesquite [20] and analysed using TNT v. 1.5 [21]. As in the original analysis by Han *et al*. [17], *Marasuchus* was set as the outgroup, and characters 2, 31, 125, 163, 196, 203, 204, 222, 227, 238, 243, 268, 292, 296, 302, 320 and 361 were ordered. Han *et al*. [17] also originally ordered characters 23, 247 and 306, but these do not represent transitional series of character states and so were not ordered here. The matrix was analysed using the new technology algorithms with a sectorial search, ratchet, drift and tree fusing using defaults and 10 random addition sequences, followed by a traditional search on the most parsimonious trees (MPTs) recovered using TBR branch swapping. All characters were equally weighted. The consistency index and retention index were calculated using the TNT script STATS.RUN. Clade support was calculated using a bootstrap, with 1000 pseudoreplicates and a New Technology search, and decay indices, which were calculated using the TNT script BREMER.RUN on a subset of 5000 MPTs.

## 3. Systematic palaeontology

Dinosauria (Owen 1842 [22])

Ornithischia (Seeley 1888 [23])

Neornithischia (Cooper 1985 [24])

*Enigmacursor mollyborthwickae* gen. et sp. nov.

**Etymology**—*Enigma*, meaning a puzzle or mystery, in reference to the convoluted taxonomic history of small-bodied ornithischians from the Morrison Formation; *cursor*, from the Latin for ‘runner’, in reference to the cursorial morphology of the elongated hind limb and pes. The species name honours Molly Borthwick, whose generous donation allowed the NHMUK to acquire the specimen.

**Holotype**—NHMUK PV R 39000, a partial skeleton that includes three teeth, three cervical, 11 dorsal, two dorsosacral and five caudal vertebrae, 10 dorsal ribs, five chevrons, right sternum, both scapulae, both humeri, both radii, both ulnae, three metacarpals, left ilium, right ischium, right pubis, both femora, both tibiae, both fibulae, right astragalus and both pedes.

**Locality and horizon**—Upper Jurassic (Kimmeridgian–Tithonian) Morrison Formation; 40°15′41″ N 108°43′38″ W, Skull Creek Estates, Moffat County, Colorado, USA. The specimen was found and excavated by Dinosaurs of America LLC in 2021−22 and acquired by David Aaron Ltd, London, UK. In 2024, the specimen was acquired by the NHMUK. The Skull Creek Estates is privately owned by Dinosaurs of America LLC: hence, no permits or licences were required for excavation or export of the specimen.

**Diagnosis**—*Enigmacursor mollyborthwickae* differs from all other ornithischian dinosaurs in possessing the following unique combination of features and one potential autapomorphy (the latter marked with an asterisk): (1) posterior articular facets offset ventrally relative to anterior articular facets on proximal dorsal vertebrae*; (2) absence of a supracetabular crest on the ilium; (3) femoral head separated from the greater trochanter by a trochanteric fossa; (4) apex of anterior trochanter situated level with the ventral margin of the femoral head; (5) absence of a ligament sulcus on the posterior surface of the femoral head; (6) ventral surface of fourth trochanter straight or slightly convex in medial or posterior view; (7) medially directed, hook-like posterior condyle of the proximal end of the tibia.

**Figure 1.**
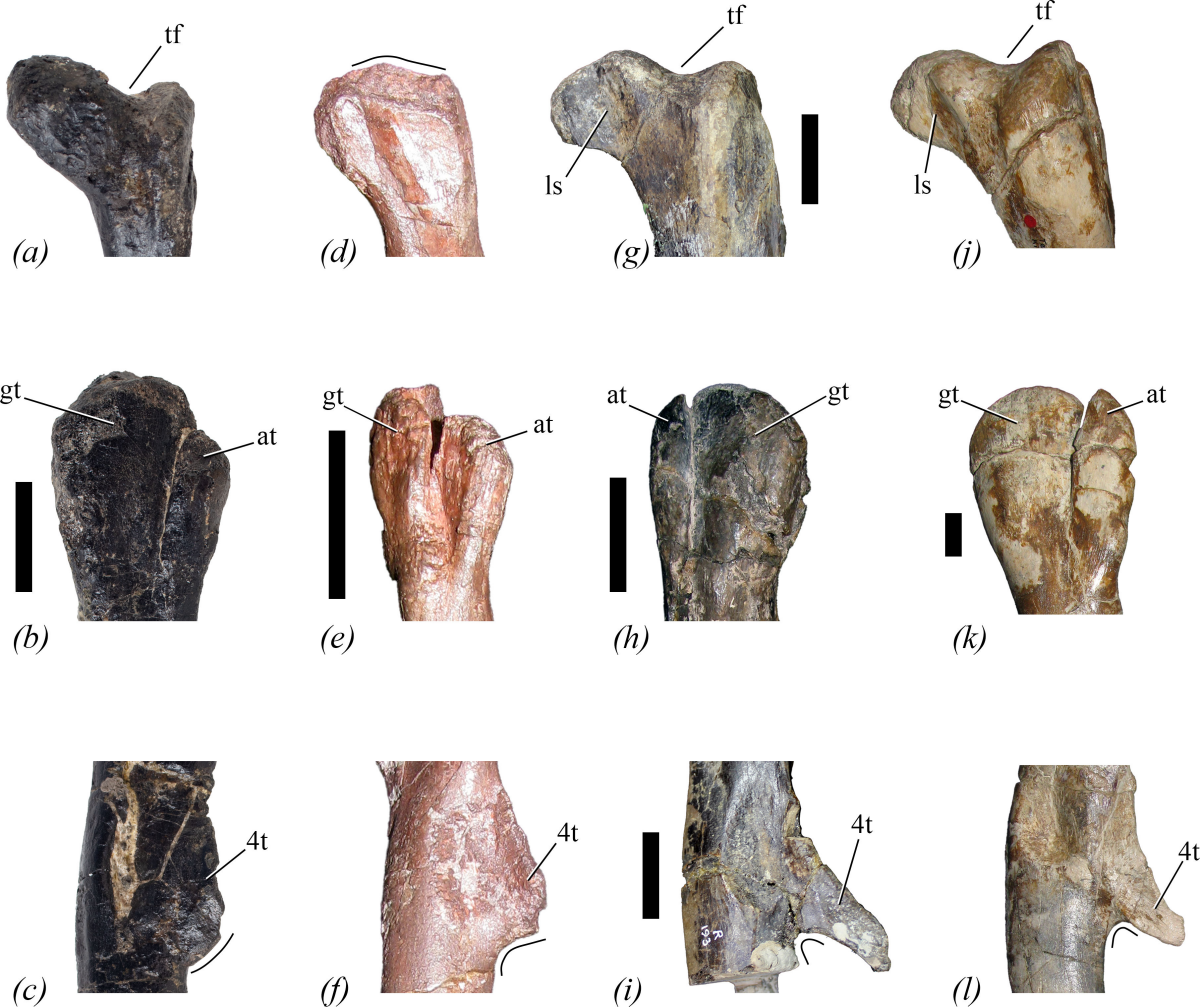
The diagnostic combination of features of the femur seen in NHMUK PV R 39000, *Enigmacursor mollyborthwickae*, in comparison to other ornithischians. (*a–c*) *Enigmacursor mollyborthwickae,* NHMUK PV R 39000, right femur; (*d–f*) *Lesothosaurus diagnosticus*, NHMUK PV RUB 17, right femur; (*g–i*) *Hypsilophodon foxii, (g*,*i*) NHMUK PV R 193, right femur; (*h*) NHMUK PV R 196, left femur; (*j–l*) *Dysalotosaurus lettowvorbecki,* MB R.2511. (*a,d,g,j*) The proximal end of the femur in posterior view showing the presence of a trochanteric fossa, tf, in all but *Lesothosaurus,* and the presence of a ligament sulcus, ls, in *Hypsilophodon* and *Dysalotosaurus* that is absent in *Enigmacursor* and *Lesothosaurus*. Note the depression on the posterior surface of the head of *Lesothosaurus* appears to be due to crushing. (*b,e,h,k*) The proximal end of the femur in lateral view showing the anterior trochanter, at, projecting lower than the greater trochanter, gt, in *Enigmacursor* and *Lesothosaurus*, but much higher in *Hypsilophodon* and *Dysalotosaurus*. Note also that the anterior trochanter is as wide anteroposteriorly as the greater trochanter in *Lesothosaurus* but it is much narrower in the other taxa. (*c,f,i,l*) Fourth trochanter, 4t, in medial view. The ventral margin of the trochanter is outwardly convex in *Enigmacursor*, but is concave in the other taxa. Although the distal end of the trochanter is broken in both *Enigmacursor* and *Lesothosaurus*, the ventral margins indicate that it could not have been pendant in the former, but probably was in the latter. Scale bars, 2 cm.

The unique combination of femoral characters is illustrated in figure 1. *Lesothosaurus diagnosticus* and *Eocursor parvus* share characters 4 and 5 with *Enigmacursor* (figure 1*e*), while *Hexinlusaurus multidens* shares these characters and character 2. However, these three taxa all lack characters 3, 6 and 7 (figure 1*d*,*f*) [25–27]. The possession of character 3 is a feature generally considered to be a neornithischian synapomorphy [28], but other early diverging neornithischians, such as *Jeholosaurus shangyuanensis*, *Haya griva* and *Hypsilophodon foxii*, lack character 4, as the anterior trochanter commonly projects much further dorsally in these taxa (figure 1*h*,*k*), and character 5, as a deep and well-developed ligament sulcus is present (figure 1*g*,*j*) [29–31]. Additionally, all these taxa have a fourth trochanter in which the ventral surface is upwardly concave (figure 1*f*,*i*,*l*), and they lack character 7. For more details about the distribution of these features, see Description.

## 4. Description

**Figure 2.**
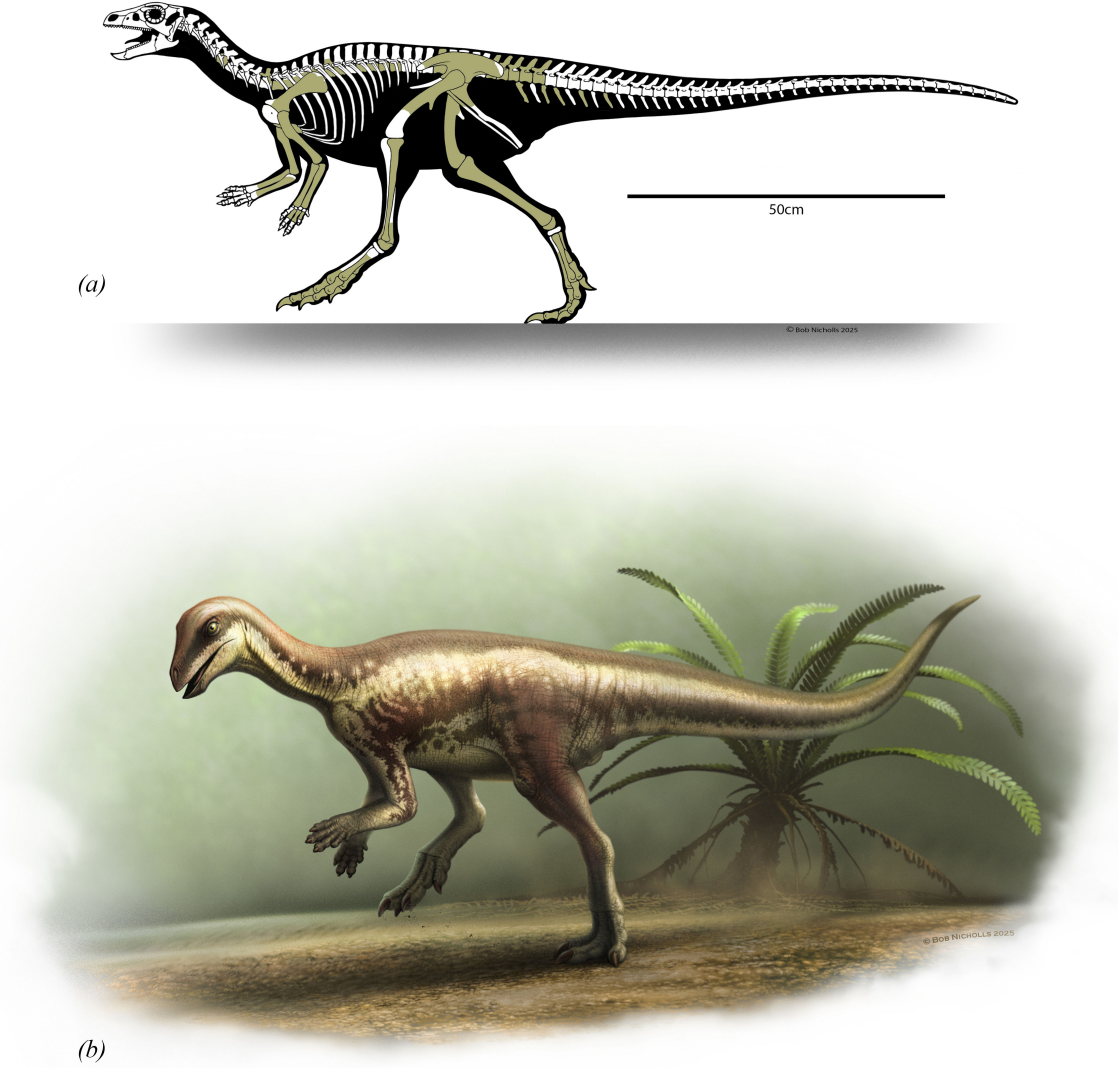
(*a*) Skeletal reconstruction of *Enigmacursor mollyborthwickae* with preserved elements shaded. (*b*) Life reconstruction of *Enigmacursor mollyborthwickae*. Artwork by Bob Nicholls.

The skeleton had been reconstructed for display prior to acquisition by the NHMUK. Missing elements were reconstructed via three-dimensional printing or modelling, while damaged elements were restored and painted. Magnets were embedded in the vertebral centra and chevrons for attachment to a mount, and armature surrounds some of the elements, preventing CT scanning of the specimen. In order to distinguish real material from reconstructed parts, radiographs of the specimen were taken at the NHMUK and these were used to inform the description and figures. The specimen was 3D surface scanned at the NHMUK and scans are available on Morphosource. Preserved parts of the skeleton are shaded in figure 2.

### 4.1. Teeth

**Figure 3.**
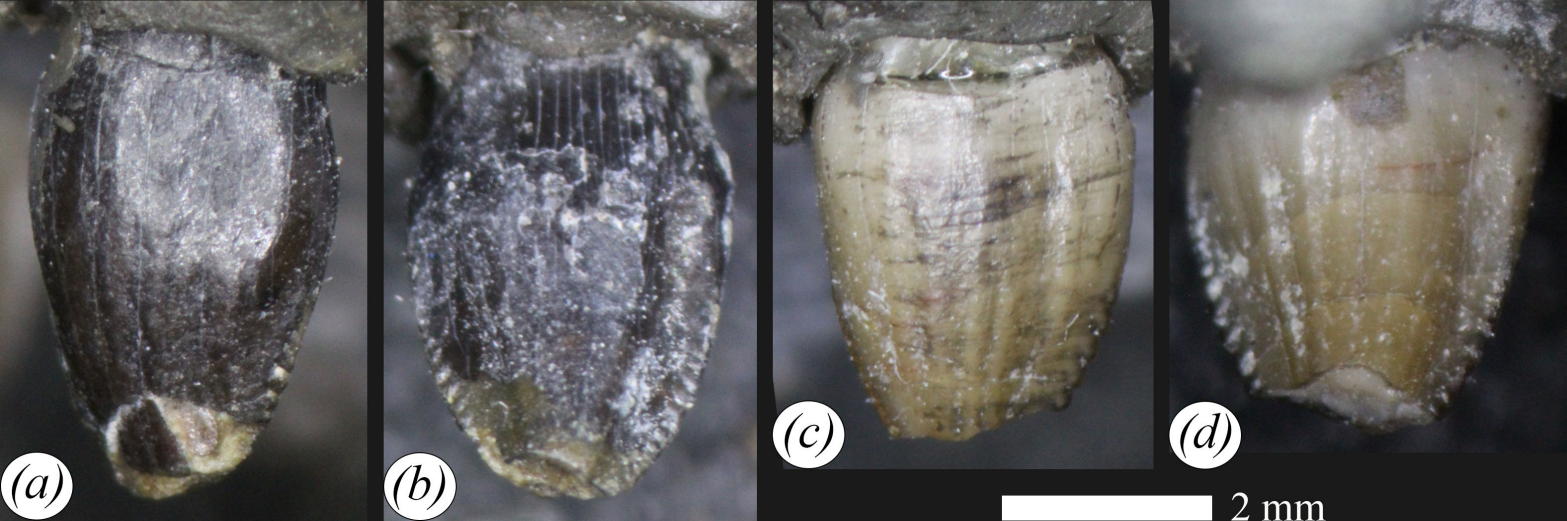
Premaxillary teeth of NHMUK PV R 39000, *Enigmacursor mollyborthwickae*, in (*a*,*c*) labial and (*b*,*d*) lingual views. Scale bar, 2 mm.

Three teeth were found in association with the postcranial skeleton, and these have been embedded in a reconstructed skull for the mount. One of the teeth is poorly preserved and retains no original morphology, while the other two are well preserved (figure 3). Comparisons with the teeth of early diverging ornithischians indicate that these are likely to be posterior premaxillary teeth.

The teeth are virtually identical in morphology. The crown bases are expanded labiolingually and mesiodistally relative to the root but lack a loph-like or ring-like cingulum. They are sub-conical in outline in labial view, similar to the premaxillary teeth of *Lesothosaurus diagnosticus* (NHMUK PV R 8501) [32] and *Jeholosaurus shangyuanensis* [33], but differing from *Agilisaurus louderbacki* [34] and *Haya griva* [30] in which they are more strongly recurved. The labial surface (figure 3*a*,*c*) is strongly convex both mesiodistally and apicobasally, and a few subtle, apicobasally extending ridges are present on the surface, but they lack the prominent ridges present in *Laquintasaura venezuelae* [35]. Both the mesial and distal margins are denticulate; one side has slightly larger denticles than the other, but because the teeth were found *ex situ*, it is not known which margin this represents. In *Orodromeus makelai* [36], the distal margin of the teeth bears the larger denticles. The posterior premaxillary teeth are denticulate in *Lesothosaurus diagnosticus* (NHMUK PV R 8501), *Agilisaurus louderbacki* [34], *Hypsilophodon foxii* [31] and *Orodromeus makelai* [36], but those of *Jeholosaurus shangyuanensis* [33] and *Haya griva* [30] lack denticles. Overall, the lingual surface (figure 3*b*,*d*) is apicobasally slightly concave but has a central portion that is mesiodistally convex, and the mesial and distal margins are drawn out into flanges separated from the central convexity by shallow sulci. This surface also has a few subtle, apicobasally extending ridges, and these are not confluent with the marginal denticles. The tips of both teeth are absent due to small, horizontal wear facets; similar facets are observed in *Lesothosaurus diagnosticus* (NHMUK PV R 8501) [37] and *Jeholosaurus shangyuanensis* [33]; this precludes the assessment of the total number of denticles on each margin.

### 4.2. Cervical vertebrae

**Figure 4.**
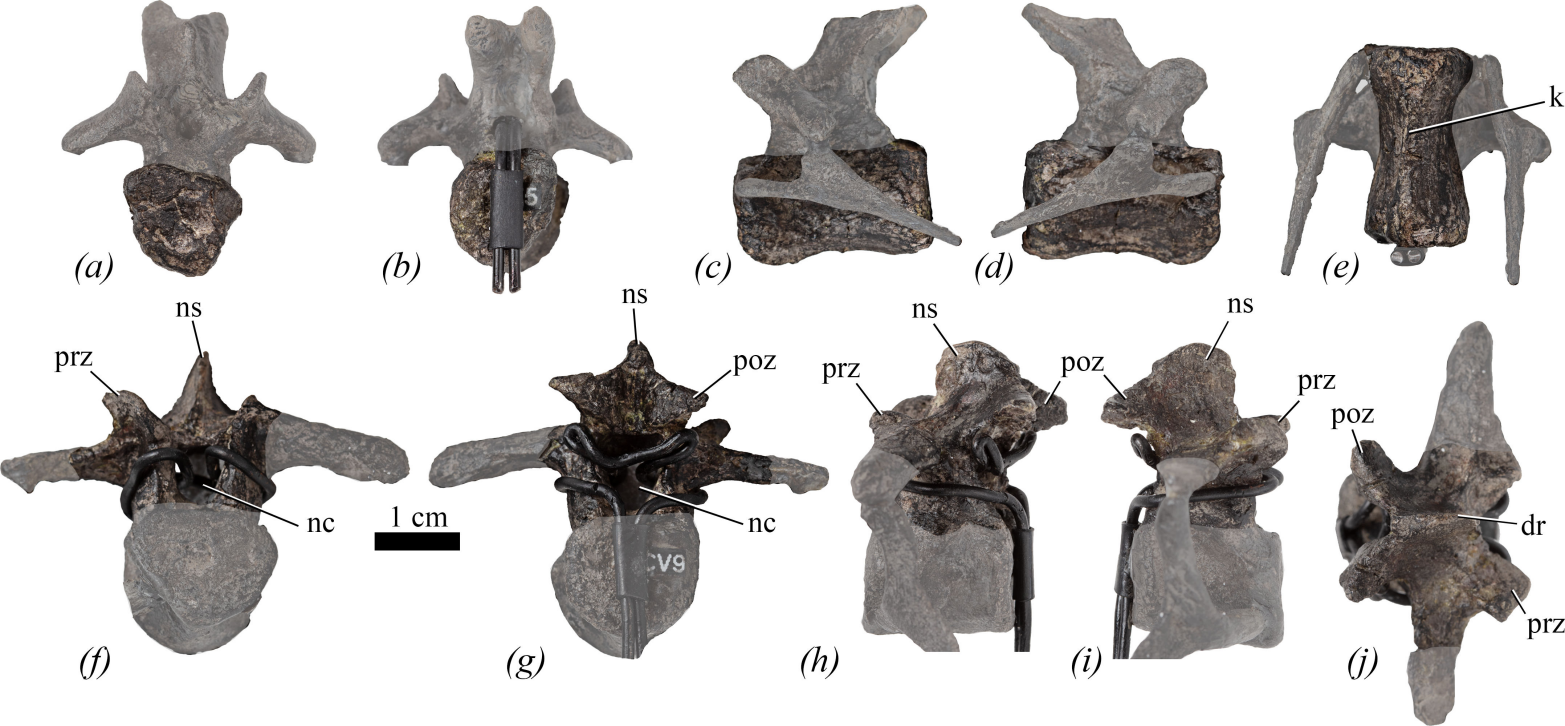
Cervical vertebrae of NHMUK PV R 39000, *Enigmacursor mollyborthwickae*, in (*a*–*e*) cervical 5; (*f*–*j*) cervical 9 in (*a*); *(f*) anterior; (*b*,*g*) posterior; (*c*,*h*) left lateral; (*d*,*i*) right lateral; (*e*) ventral; and (*j*) dorsal views. dr, dorsal ridge; k, keel; nc, neural canal; ns, neural spine; poz, postzygapophysis; prz, prezygapophysis. The greyed out area indicates reconstruction. Reconstructed ribs have been cropped from the images for clarity in (*a,b,f,g*,*j*). Scale bar, 1 cm.

Three cervical vertebrae (Cv) are preserved (figure 4). For convenience, the cervical vertebrae are numbered in the positions in which they are mounted, but their true positions are unknown. Two of the vertebrae are represented only by centra (Cv5 and Cv7), while the third preserves only the neural arch (Cv9). The posterior surfaces of the vertebrae are partially obscured by armature. Radiographs of the vertebrae did not reveal any internal cavities in the centra or the neural arches.

The centra of Cv5 (figure 4*a*–*e*) and Cv7 are longer anteroposteriorly than they are wide mediolaterally or tall dorsoventrally (see table 1 for measurements of all vertebrae). This appears similar to the condition in *Hexinlusaurus multidens, Yandusaurus hongheensis* [27], *Minimicursor phunoiensis* [38], *Sanxiasaurus modaoxiensis* [39], *Camptosaurus dispar* [40] and *Iani smithi* [41], but contrasts with the condition in *Haya griva* [30], *Jeholosaurus shangyuanensis* [29], *Orodromeus makelai* [36], *Yueosaurus tiantaiensis* [42], *Tenontosaurus tilletti* [43] and *Camptosaurus aphanoecetes* [40] in which the centra are approximately as long as they are tall, and *Mahuidacursor lipanglef* [44] and *Talenkauen santacrucensis* [45] in which they are very elongated relative to their height. The lateral surfaces are smoothly continuous with the ventral surface and are not offset from the latter by distinct breaks-in-slope. In lateral view (figure 4*c*,*d*), the lateral surfaces are saddle-shaped (anteroposteriorly concave and slightly convex dorsoventrally) similar to most ornithischians but in contrast to the condition in *Haya griva* [30] and *Sanxiasaurus modaoxiensis* [39], where there are fossae on the lateral surfaces of the centra. The parapophyses are obscured by the reconstructed cervical ribs, but small swellings that probably represent these facets are positioned on the lateral surfaces, immediately posterior to the anterior articular facet, close to the presumed location of the neurocentral suture (the exact position of the neurocentral suture is unclear because of the reconstructed neural arches). In ventral view, the centrum of Cv5 bears a low midline keel that extends for the full length of the centrum (figure 4*e*,*k*) and remains narrow along its entire length. By contrast, in Cv7 a short, faint midline keel is restricted to the middle of the centrum, but anteriorly this expands transversely to form a rugose platform immediately ventral to the anterior articular surface. This platform forms the ventral border of an anteroposteriorly short, shallow excavation on the ventrolateral corner of the lateral surface. This variation in midline keel morphology differs from the condition in *Eocursor parvus* [26], *Hexinlusaurus multidens, Yandusaurus hongheensis* [27], *Haya griva* [30], *Hypsilophodon foxii* [31], *Iani smithi* [41], *Dryosaurus elderae* (CM 11 340, CM 87 688, CM 3392), *Mahuidacursor lipanglef* [44], *Tenontosaurus tilletti* [43] and *Camptosaurus aphanoecetes* [40], in which a prominent keel is consistently present for the full length of the centrum in all cervical vertebrae. However, without preservation of the entire cervical series, the taxonomic significance of this feature is equivocal.

**Table 1.** Measurements of vertebrae of NHMUK PV R 39000, *Enigmacursor mollyborthwickae*. *Includes the height of the chevron facet.

vertebral number	anteroposterior length of centrum (mm)	maximum transverse width of anterior articular surface (mm)	maximum dorsoventral height of anterior articular surface (mm)	maximum transverse width of posterior articular surface (mm)	maximum dorsoventral height of posterior articular surface (mm)
Cv5	23.2	13.4	12.1	12.7	11.7
Cv7	22.8	15.4	12.1	14.7	13.8
D1	17.8 (as preserved)	15.2	11.7	15.1	11.9
D2	20.0	14.2	17.2	15.9	18.4
D3	18.4	15.7	14.9	15.1	15.0
D4	23.3	16.1	18.2	15.4	17.6
D5	18.6	15.0	16.8	13.9	18.7
D6	21.3	14.9	15.4	13.8	14.4
D7	20.8	16.7	17.9	17.8	19.5
D9	23.3	16	19.3	16	17.6
D10	21.1	18.4	19.1	16	20.4
D11	26.0	19.0	21.0	19.4	19.9
D12	24.8	18.1	21.5	17.9	19.7
DS1	22.2	19.7	16.3	20.2	16.0
DS2	23.5	18.2	16.8	20.3	16.8
CD1	20.2	12.4	18.4	13.6	19.6*
CD2	22.1	15.0	16.3	14.0	21.3*
CD3	22.3	14.4	18.4	15.4	22.1*
CD4	21.8	19.8	19.0	19.2	17.6*
CD5	20.7	20.4	14.7	17.3	19.7*

In both Cv5 and Cv7, the anterior articular facets (figure 4*a*) are shield-shaped with a straight dorsal margin, ventrally converging straight lateral margins and either a straight (Cv7) or slightly rounded (Cv5) ventral margin. The anterior articular surfaces are flat or very subtly concave, similar to other non-iguanodontian ornithischians (e.g. *Eocursor parvus* [26], *Hexinlusaurus multidens* [27], *Haya griva* [30], *Jeholosaurus shangyuanensis* [29], *Mahuidacursor lipanglef* [44], *Thescelosaurus neglectus* [46]) and *Dryosaurus elderae* (CM 87688), whereas in *Camptosaurus* [40], *Hypsilophodon foxii* [31], *Cumnoria prestwichii* [47] and *Talenkauen santacrucensis* [45], they are slightly convex. In *Enigmacursor*, the anterior articular facets are slightly wider mediolaterally than they are tall dorsoventrally. The posterior articular facet of Cv5 (figure 4*b*) is slightly distorted laterally, so its original outline is unclear, and is flat, whereas that of Cv7 has a sub-circular outline and is gently concave. Gently concave posterior articular facets are common in non-iguanodontian ornithischians, e.g. *Eocursor parvus* [26], *Haya griva* [30], *Jeholosaurus shanyuanensis* [29], *Mahuidacursor lipanglef* [44], but are more deeply concave in iguanodontians such as *Dryosaurus elderae* (CM 87 688) and *Talenkauen santacrucensis* [45]. No foramina are present in *Enigmacursor* and vertebral laminae and fossae are absent.

The neural arch of Cv9 is fairly complete but lacks the diapophyses and the tip of the left prezygapophysis (figure 4*f*–*j*). In anterior and posterior views, the neural canal is semicircular in outline with a straight dorsal margin, although the aperture is larger in posterior view (figure 4*f*,*g*, nc). In lateral view, the prezygapophyses (figure 4*h*,*i*, prz) extend anteriorly from the anterolateral corners of the neural arch platform and extend to a point level with the anterior articular facet. They are separated by a wide, ‘U’-shaped gap in dorsal view (figure 4*j*). Their articular surfaces are oriented dorsomedially and have a sub-rectangular outline. A ridge extends dorsally along the midline between the prezygapophyses and is confluent with the anterior margin of the neural spine (figure 4*j*, dr). A similar ridge is also observed in *Iani smithi* [41]. The neural spine is plate-like and transversely compressed. In lateral view (figure 4*h*,*i*, ns), the neural spine extends further dorsally than the postzygapophyses, is anteroposteriorly expanded and its summit is dorsoventrally convex (although this might have been altered by breakage and/or slight reconstruction). The development of the neural spine is similar to the condition in early diverging neornithischians (e.g. *Jeholosaurus shangyuanensis* [29], *Orodromeus makelai* [36], *Iani smithi* [41], *Thescelosaurus neglectus* [46], *Minimocursor phunoiensis* [38]), but differs from that in some early diverging iguanodontians, such as *Dryosaurus elderae* (CM 87688, CM 3392), *Camptosaurus* spp. [40,48] and *Mahuidacursor lipanglef* [44] in which cervical vertebrae do not have distinct neural spines that project above the postzygapophyses. In *Tenontosaurus tilletti* [43] and *Changmiania liaoningensis* [49], the neural spines project dorsal to the postzygapophyses, but they are triangular in lateral view, rather than plate-like. In dorsal view, the posterior margin of the neural spine of *Enigmacursor* bifurcates to form two ridges that extend to the postzygapophyses (figure 4*j*, poz), forming their dorsal margin, as in *Iani smithi* [41]. The postzygapophyses are separated by a wide, ‘V’-shaped cleft in both dorsal and posterior views (figure 4*g*,*j*, poz). In lateral view, the postzygapophyses extend slightly posterior to the posterior articular facet (figure 4*h*,*i*, poz), and are relatively short, similar to the postzygapophyses of *Eocursor parvus* [26], *Orodromeus makelai* [36] and *Jeholosaurus shangyuanensis* [29], but differing from the elongated postzygapophyses of *Hypsilophodon foxii* [31], *Dryosaurus altus* [50], *Camptosaurus* [40], *Mahuidacursor lipanglef* [44], *Tenontosaurus tilletti* [43], *Iani smithi* [41], *Thescelosaurus neglectus* [46] and *Talenkauen santacrucensis* [45]. Their articular surfaces are angled ventrally, slightly laterally and are sub-elliptical in outline, with the longest axis extending posterolaterally. No other vertebral laminae are present.

### 4.3. Dorsal vertebrae

**Figure 5.**
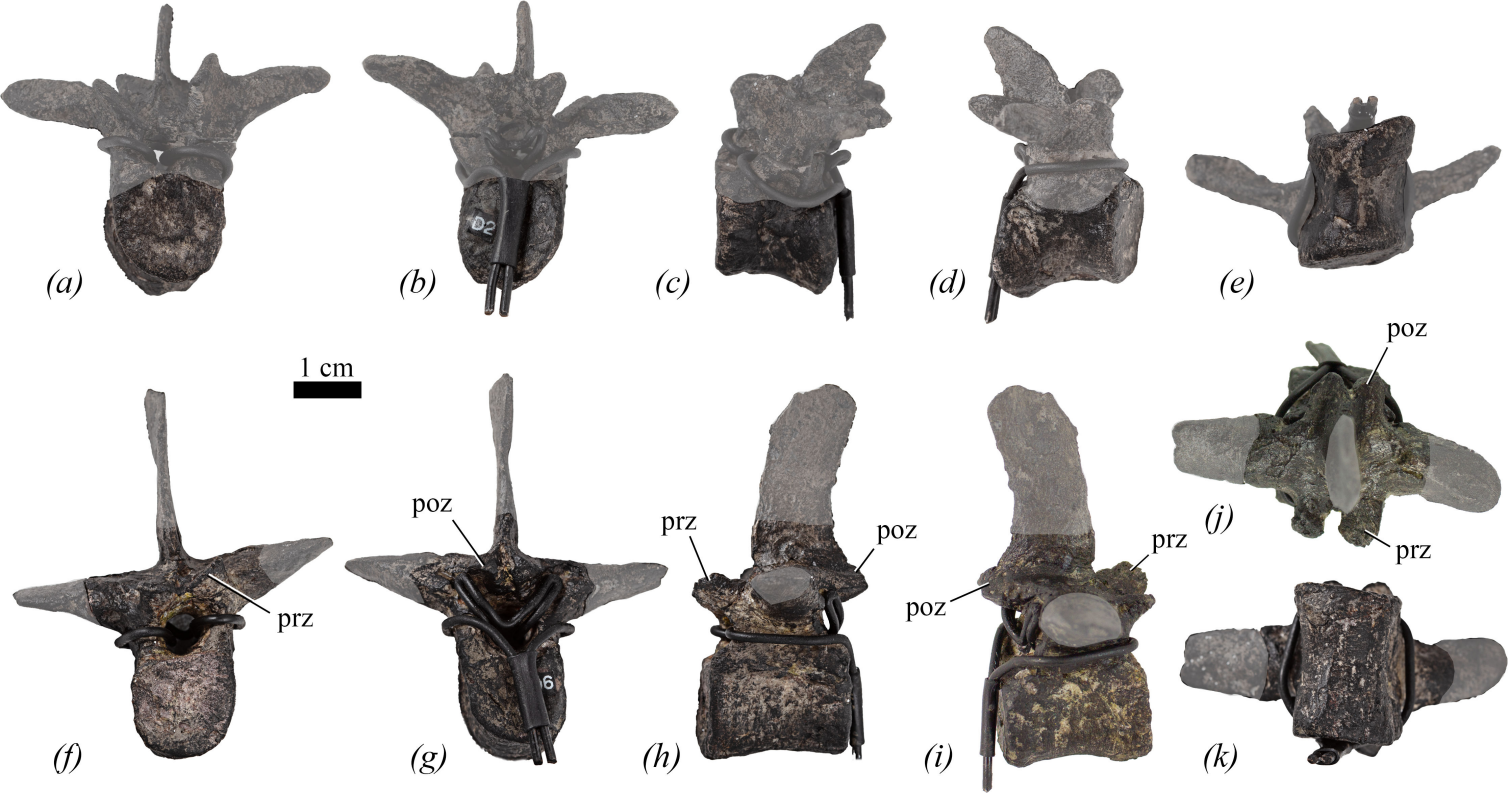
Dorsal vertebrae of NHMUK PV R 39000, *Enigmacursor mollyborthwickae*, in (*a*–*e*) dorsal 2; (*f*–*k*) dorsal 6 in (*a*); (*f*) anterior; (*b,g*) posterior; (*c,h*) left lateral; (*d,i*) right lateral; (*e,k*) ventral; and (*j*) dorsal views. poz, postzygapophysis; prz, prezygapophysis. The greyed out area indicates reconstruction. Scale bar, 1 cm.

In figure 5, the mounted skeleton is reconstructed with 15 dorsal vertebrae. Again, for convenience, they are numbered as mounted, but in the absence of detailed quarry notes it is not clear if this sequence is correct, although their proportions suggest that the sequence is a reasonable approximation if not entirely accurate. D8 and D13−15 are completely reconstructed. Neural spines are reconstructed on all vertebrae except for D5 and D6, in which the bases of the neural arches are preserved, and in D9, where there is a partial (but very poorly preserved) neural arch. D1−3 may be transitional ‘cervicodorsal’ or ‘pectoral’ vertebrae, but poor preservation makes it difficult to determine the exact boundary between the neck and trunk. The posterior surfaces of all the dorsals are partially obscured by armature.

In lateral view, the centra of D1−3 gradually increase in anteroposterior length along the series (table 1). These centra differ from more posterior dorsals in having sub-trapezoidal lateral outlines due to a distinct offset between the heights of the anterior and posterior articular surfaces, with the posterior surfaces being dorsoventrally taller than the anterior surfaces (figure 5*c*,*d*). This offset of the anterior and posterior facets appears to be unusual and potentially autapomorphic. Although a similar offset is commonly seen in the anterior cervical vertebrae of neornithischians (e.g. *Hypsilophodon foxii* [31], *Tenontosaurus tilletti* [43], *Camptosaurus* spp. [40,48]), it has not been previously reported in the dorsal series, although it might be present in *Iani smithi* [41, fig. 150]. The ventral margins are more noticeably upwardly concave in the anterior dorsals than in the more posterior dorsal vertebrae. The lateral surfaces of the centra are saddle-shaped (anteroposteriorly concave and dorsoventrally convex) and lack laminae, fossae, foramina and any evidence of parapophyses (figure 5*c*,*d*). The lateral surfaces converge ventrally, merging to form a narrow, keel-like ventral margin in D1; however, in D2 and D3, the ventral surfaces are expanded transversely and lack a keel, but they are not offset from the lateral surfaces by a distinct break-in-slope (figure 5*e*). The ventral surfaces of the anterior dorsal vertebrae differ from those of *Changchunsaurus parvus* [51] and *Jeholosaurus shangyuanensis* [29] as the latter taxa possess a swelling with a rugose surface texture on the anterior centrum ventral margin, which is absent in *Enigmacursor*.

D1 is slightly distorted transversely. In anterior and posterior views, the centrum has transversely narrow, elliptical, flat articular surfaces that are dorsoventrally taller than they are transversely wide. In D2, the anterior articular surface is flat and is only slightly deeper dorsoventrally than wide transversely (figure 5*a*). It has a square outline with almost straight lateral and dorsal margins and a gently convex ventral margin. That of D3 is identical in morphology to D2 but is sub-equal in transverse and dorsoventral dimensions. In posterior view, the articular surfaces of D2 and D3 have sub-circular outlines and are very shallowly concave (figure 5*b*).

In lateral view, D4 has a rectangular outline, lacks the ventral concavity seen in D1−3 and there is no offset between the anterior and posterior articular facets, but is otherwise identical in morphology. It is slightly crushed, which may have affected the shape of its anterior articular surface. In anterior view, the articular surface is flat and has a shield-shaped outline that narrows ventrally. The posterior articular surface has a similarly shaped outline but is gently concave.

D5−7 and D9−12 have centra that are essentially identical in lateral view, differing only in size (figure 5*f*–*k*). The lateral surfaces are saddle-shaped and continuous with the ventral margin, lacking any distinct break in slope between them (figure 5*h*,*i*). Small elliptical nervous or vascular foramina are frequently present at centrum midlength close to the ventral margins, as also occurs in *Jeholosaurus shangyuanensis* [29], *Changchunsaurus parvus* [51], *Parksosaurus warreni* [52] and *Cumnoria prestwichii* [47] but in contrast to *Haya griva,* where such foramina are absent [30]. Most of the dorsal centra are longer anteroposteriorly than they are tall dorsoventrally, except for D5, which is equidimensional. None of the centra bear ventral midline keels or sulci (figure 5*k*), similar to *Dryosaurus elderae* (CM 11340, CM 87688, CM 3392), *Hypsilophodon foxii* [31], *Camptosaurus dispar* [40] and *Cumnoria prestwichii* [47], but differing from the condition in *Haya griva* [30], *Jeholosaurus shangyuanensis* [29], *Orodromeus makelai* [36], *Camptosaurus aphanoecetes* [40] and *Mahuidacursor lipanglef* [44] in which the posterior dorsals are weakly keeled, and in *Parksosaurus warreni* [52] in which there is a sulcus. Anterior articular surfaces are sub-circular to sub-quadrate in outline and are approximately as tall as they are wide. All anterior and posterior articular surfaces are flat or very slightly concave (figure 5*f*,*g*).

The base of the neural arch is present in D5 and D6 (figure 5*f*–*j*), but in D5 the neural arch platform is entirely obscured by the reconstruction of the vertebral processes. In D6, the prezygapophyses are preserved. In lateral view (figure 5*h*,*i*, prz), they extend slightly anterior to the centrum and are orientated almost horizontally, while in dorsal view they are separated by a narrow ‘U’-shaped cleft and have sub-elliptical articular facets that face dorsally and slightly medially (figure 5*j*). The neural arch of D9 is too poorly preserved to offer useful anatomical information.

### 4.4. Posterior dorsal or dorsosacral vertebrae

**Figure 6.**
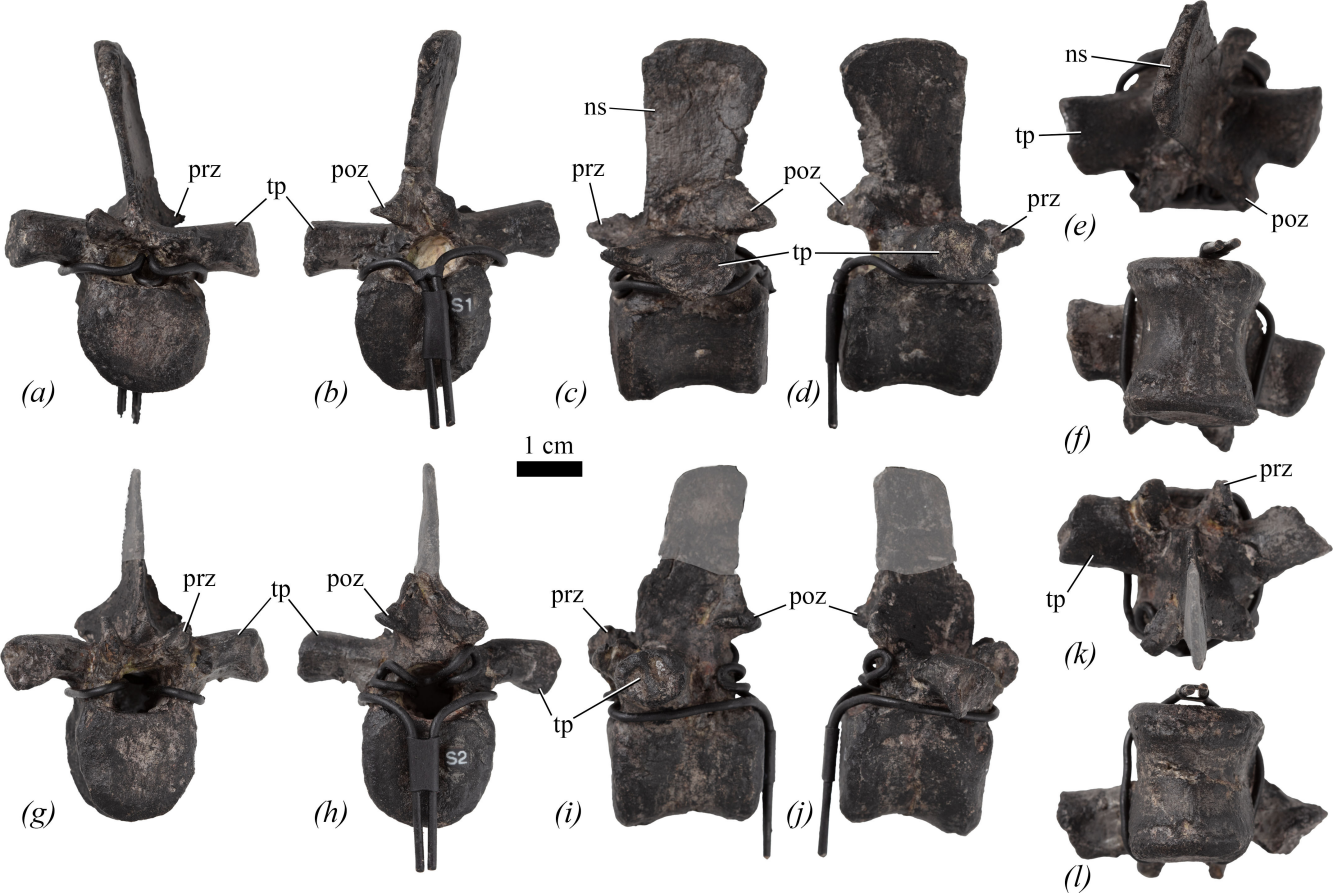
Dorsosacral vertebrae of NHMUK PV R 39000, *Enigmacursor mollyborthwickae*, in (*a–f*) dorsosacral 1; (*g–l*) dorsosacral 2 in (*a*); (*g*) anterior; (*b,h*) posterior; (*c,i*) left lateral; (*d,j*) right lateral; (*e,k*) dorsal; and (*f,l*) ventral views. ns, neural spine; poz, postzygapophysis; prz, prezygapophysis; tp, transverse process. The greyed out area indicates reconstruction. Scale bar, 1 cm.

Two vertebrae are mounted as sacrals 1 and 2, but they exhibit neither signs of fusion to each other nor sacral ribs, and their proportions are similar to the dorsals (figure 6; table 1). The presence of a combined facet for a single-headed rib (see below) indicates these are likely to be the most posterior dorsals or perhaps dorsosacrals. We refer to these vertebrae as dorsosacrals (DS) for convenience.

Both vertebrae are almost complete, lacking only small parts of the neural arches, and some of the surfaces have been partially reconstructed/painted over. The vertebrae are essentially identical to each other. The centra are amphiplatyan, with saddle-shaped lateral surfaces that are pierced by small nutrient foramina at approximately midlength, as in the other dorsal vertebrae (figure 6*c*,*d*,*i,j*). In ventral view, the centra are waisted and spool-like (figure 6*f*,*l*). The lateral surfaces are separated from the ventral surface by a subtle break in slope but not by distinct lateral ridges. There is neither a ventral keel nor groove. The centra are slightly longer than tall in lateral view with a gently concave ventral margin. In anterior and posterior views, the centra have sub-circular articular facets (figure 6*a*,*b*,*g*,*h*). The junction between the articular surfaces and lateral surfaces is subtly ornamented with numerous short longitudinal striations. Similar striations are common on the dorsal vertebrae of a range of neornithischian taxa, including *Changchunsaurus parvus* [51], *Haya griva* [30], *Iani smithi* [41] and *Cumnoria prestwichii* [47].

In lateral view, both the pre- and postzygapophyses project only a small distance beyond the anterior/posterior surface of the centrum, respectively (figure 6*c*,*d*,*i*,*j*, prz, poz). In DS1, the prezygapophyses have a sub-triangular outline in lateral view that tapers anteriorly, whereas in DS2 the anterior margins are rounded. In dorsal view, the prezygapophyses are separated by a broad ‘U’-shaped groove (figure 6*e*,*j*, prz). The articular facets are oriented dorsally and slightly medially in DS1 but more vertically in DS2. In dorsal view, the transverse processes have sub-parallel anterior and posterior margins, and in DS1 extend strictly laterally, whereas they are slightly anterolateral in DS2 (figure 6*e*,*j*, tp). They are sub-elliptical in longitudinal cross section with a long axis extending anteroposteriorly, and their articular surfaces retain the same elliptical outline. There is no indication of a separate parapophysis, so it is assumed that it is merged with the diapophysis, and that the ribs were single-headed in this part of the dorsal vertebral column. A merged para- and diapophysis is also seen on D13 and more posteriorly in many neornithischians including *Changchunsaurus* [51], *Thescelosaurus assiniboiensis* [53], *Orodromeus makelai* [36], *Hypsilophodon foxii* [31] and *Tenontosaurus tilletti* [43]. In anterior view, the transverse processes extend horizontally (figure 6*a*,*g*, tp). The postzygapophyses are positioned slightly dorsally to the prezygapophyses. They are blunt triangular processes in lateral view and their articular surfaces face ventrally and slightly laterally. In dorsal view, they are separated by a shallow ‘U’-shaped sulcus. The neural spine of DS1 is complete while that of DS2 is missing its dorsal-most half (figure 6, ns). In lateral view, the neural spine has straight anterior and posterior margins which are sub-parallel to each other so that the spine increases slightly in anteroposterior length dorsally (figure 6*c*,*d*, ns). It has a gently convex dorsal margin. In anterior view, the neural spines are laterally compressed plates. In anterior or posterior view, the neural canal has a sub-circular outline.

### 4.5. Dorsal ribs

Parts of 10 dorsal ribs are preserved, but none are complete, and most comprise broken fragments of rib shafts. These are generally rod-like, but detailed morphological description is impossible due to reconstruction.

### 4.6. Caudal vertebrae

**Figure 7.**
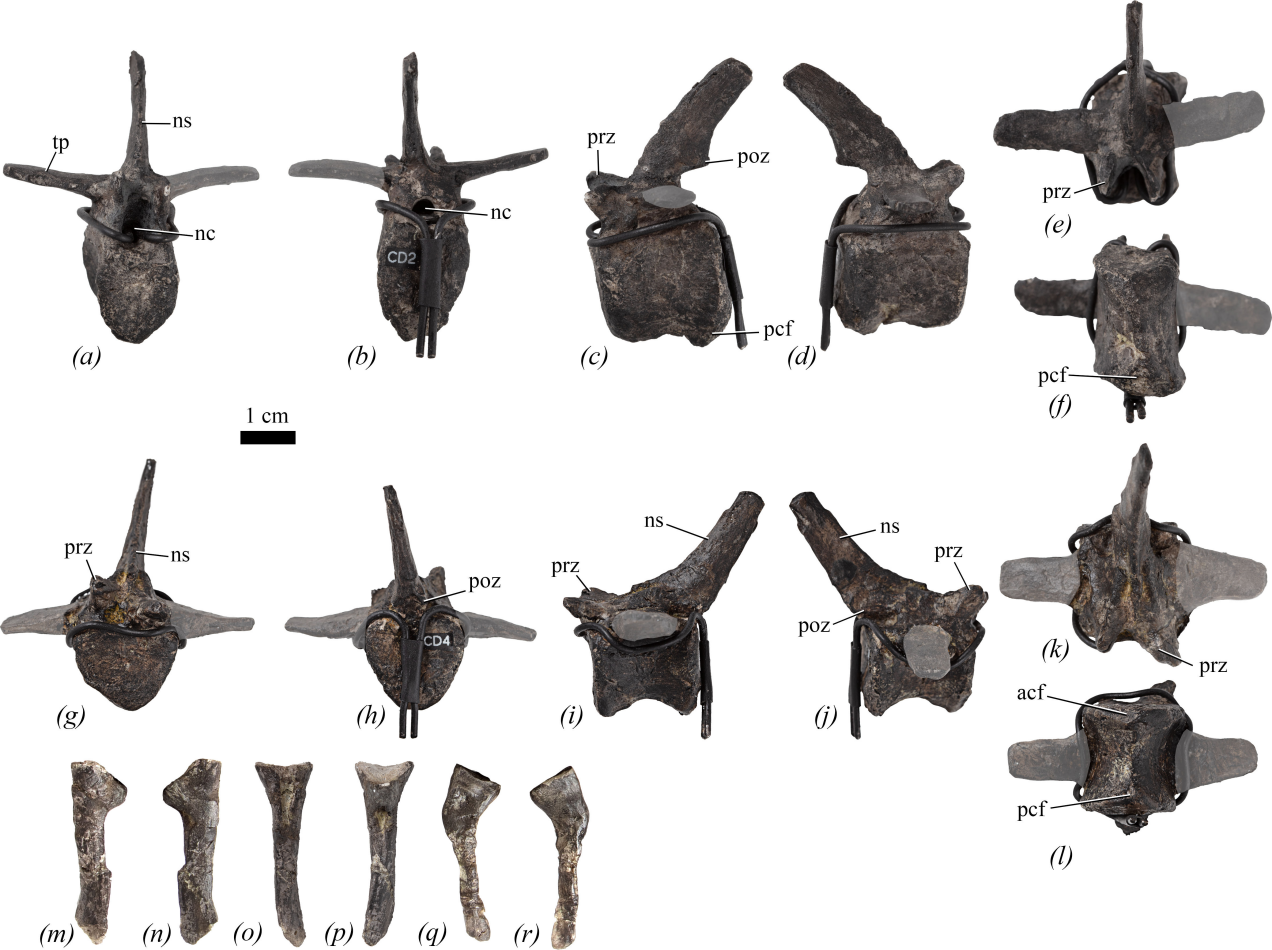
Caudal vertebrae and chevrons of NHMUK PV R 39000, *Enigmacursor mollyborthwickae*, in (*a–f*) caudal 2; (*g–l*) caudal 4; (*m–r*) two chevrons, location in the tail unknown in (*a,g,o*) anterior; (*b,h*,*p)* posterior; (*c,i*,*m,q*) left lateral; (*d,j*,*n,r*) right lateral; (*e,k*) dorsal; and (*f,l*) ventral views. acf, anterior chevron facet; nc, neural canal; ns, neural spine; pcf, posterior chevron facet; poz, postzygapophysis; prz, prezygapophysis; tp, transverse process. The greyed out area indicates reconstruction. Scale bar, 1 cm.

Only Cd1−5 are preserved (figure 7). The vertebra labelled as Cd5 lacks chevron facets and could be either a dorsal or a caudosacral centrum. The neural spine of Cd5 is entirely reconstructed and its centrum is identical to those of the dorsosacrals (see §4.4).

In lateral view, the centra of Cd1−4 are slightly longer anteroposteriorly than tall dorsoventrally (table 1; figure 7*c*,*d*,*i*,*j*), similar to *Jeholosaurus shangyuanensis* [29], but in contrast to those of *Parksosaurus warreni* [52] and *Haya griva* [30] in which the proximal caudals are shorter than they are tall. In lateral view, the anterior and posterior margins of the centra are straight and the ventral margin is gently concave (figure 7*c*,*d*,*i*,*j*). The lateral surfaces of the centra are saddle-shaped and converge ventrally. They lack foramina, as in *Hexinlusaurus multidens* [27] and *Haya griva* [30], but unlike the caudal vertebral centra of *Dryosaurus elderae* (CM 21 786) and *Jeholosaurus shangyuanensis* [29] in which many foramina are present. The centra of Cd1−4 lack a distinct ventral surface, and ventral grooves and keels are absent (figure 7*f*,*l*), as in *Haya griva* [30]. This contrasts with the condition in *Dryosaurus elderae* (CM 21 786), *Parksosaurus warreni* [52], *Hexinlusaurus multidens* [27], *Orodromeus makelai* [36], *Valdosaurus canaliculatus* [54] and a large specimen of *Jeholosaurus shangyuanensis* [29] in which keels extend from the anterior to posterior chevron facets in the proximal caudal vertebrae. However, the absence of a keel in a smaller specimen of *Jeholosaurus* led Han *et al*. [29] to conclude that the presence/absence of this feature could be ontogenetic. In *Enigmacursor*, the posteroventral margins of the centra are bevelled due to the presence of chevron facets. In anterior or posterior view, the articular surfaces of Cd1−3 are taller dorsoventrally than wide transversely (table 1; figure 7*a*,*b*), with narrow sub-triangular to sub-elliptical outlines, whereas in Cd4, the articular facets have sub-equal transverse and dorsoventral diameters and a rounded outline (figure 7*g*,*h*). The articular facets of all four centra are flat to very slightly concave. Cd1−3 lack a distinct anterior chevron facet, although a small rugose area is present in this position in all three (figure 7*f*). By contrast, all these centra bear a distinct, sub-crescentic posterior chevron facet (figure 7, pcf).

Partial neural arches are available for Cd1−4, but none possess a complete neural spine or transverse processes. In lateral view, the prezygapophyses extend a short distance beyond the anterior articular facet and are angled at approximately 45° to the horizontal (figure 7, prz). They are similarly developed to those of *Hexinlusaurus multidens* [27], *Jeholosaurus shangyuanensis* [29] and *Iani smithi* [41], but in *Parksosaurus warreni* [52], *Hypsilophodon foxii* [31], *Haya griva* [30], *Tenontosaurus tilletti* [43] and *Camptosaurus aphanoecetes* [40] the prezygapophyses project further anteriorly and are more robust than in *Enigmacursor*. In dorsal view, the prezygapophyses have blunt, rounded tips separated by a shallow ‘U’-shaped groove, and have articular facets that face almost entirely medially (figure 7*e*,*k*), as in *Parksosaurus warreni* [52], *Hexinlusaurus multidens* [27] and *Tenontosaurus tilletti* [43]. What remains of the transverse processes indicates that they had sub-parallel anterior and posterior margins in dorsal view (figure 7*e*) and a narrow elliptical cross section with the long axis trending anteroposteriorly. This contrasts with the condition in *Iani smithi* [41] and *Thescelosaurus neglectus* [46] in which the transverse processes are much deeper dorsoventrally on the proximal caudal vertebrae. The transverse processes are situated at the level of the neural arch boundary. Postzygapophyses are preserved in Cd2 (figure 7, poz). They are positioned at approximately the same level as the prezygapophyses, at the base of the neural spine, and are small oval facets that face ventrolaterally. The neural spine is anteroposteriorly narrow in lateral view, is transversely compressed and extends posterodorsally (figure 7, ns). The neural spines of *Iani smithi* [41], *Jeholosaurus shangyuanensis* [29], *Changmiania liaoningensis* [49] and *Tenontosaurus tilletti* [43] appear to extend much more vertically, those of *Iani smithi* [41], *Tenontosaurus tilletti* [43] and *Camptosaurus aphanoecetes* [40] are proportionately longer, while those of *Parksosaurus warreni* [52], *Minimocursor phunoiensis* [38], *Changmiania liaoningensis* [49] and *Thescelosaurus neglectus* [46] are anteroposteriorly broader and more plate-like. In anterior or posterior view, the neural canals are sub-elliptical with the long axis extending dorsoventrally (figure 7, nc).

### 4.7. Chevrons

Five chevrons are preserved. They are straight in anterior (figure 7*o*) and lateral (figure 7*m*,*n*,*q*,*r*) views, with a proximal expansion. The proximal end is more expanded transversely than anteroposteriorly; the original shape of the proximal surface is unknown because all are covered in plaster in this area with magnets embedded for attachment to the mount. Ventral to the proximal expansion, in lateral view, the anterior and posterior margins of the shaft are parallel to each other, and the shafts are transversely compressed, being wider anteroposteriorly than they are transversely. The distal ends are missing in all cases. In anterior view, the chevron is Y-shaped, tapering distally, with a matrix-filled haemal canal between the two upper branches of the ‘Y’ (figure 7*o*,*p*). Dorsally, the branches meet on the midline, but it is unclear if they were fused due to reconstruction of the proximal end. Posteriorly, the two upper branches of the ‘Y’ form sharp ridges that taper distally below the haemal canal. The chevrons appear to be similar in morphology to those of other small-bodied ornithischians, such as *Hypsilophodon foxii* [31], *Orodromeus makelai* [36] and *Haya griva* [30].

### 4.8. Scapula

**Figure 8.**
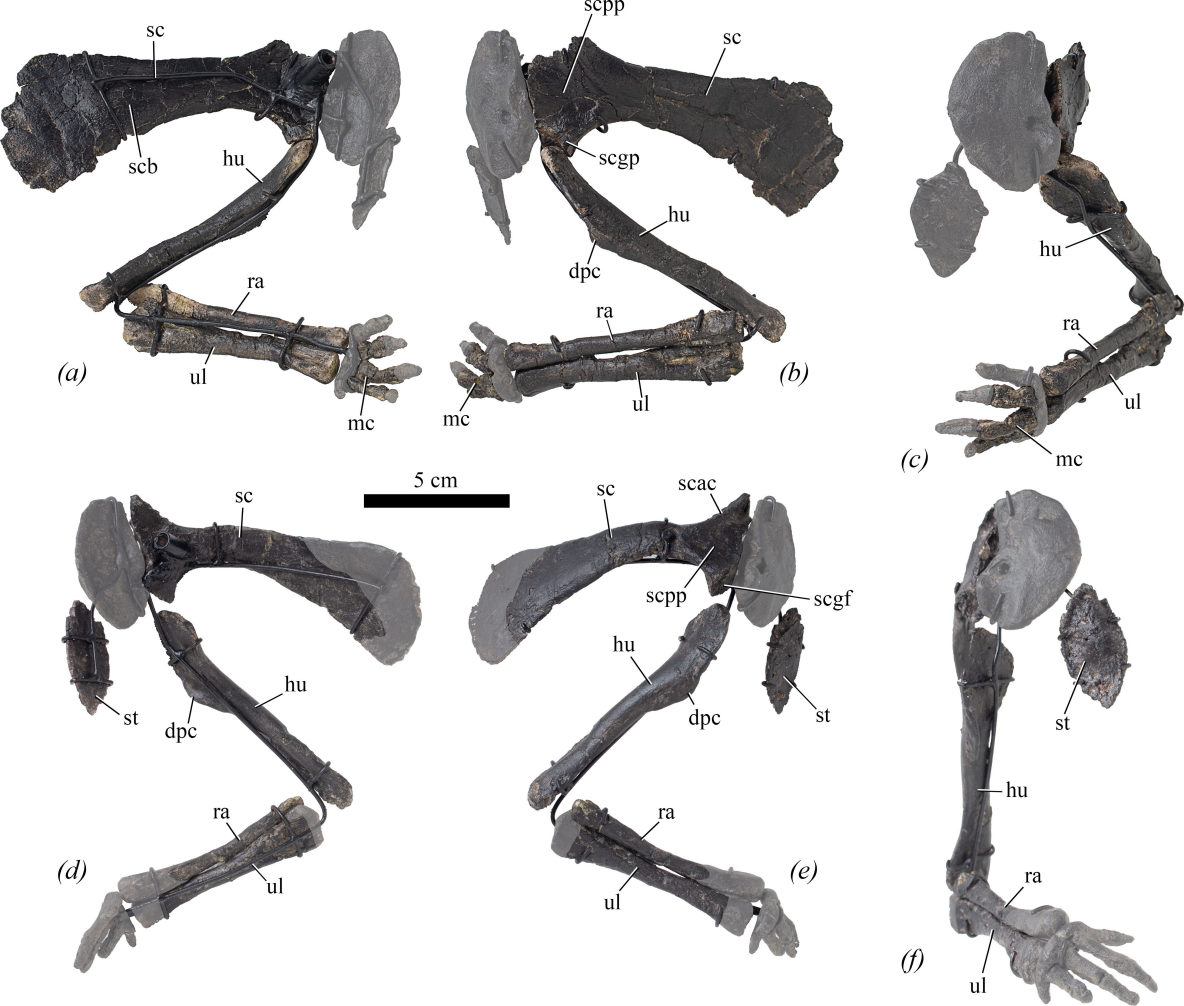
Forelimbs of NHMUK PV R 39000, *Enigmacursor mollyborthwickae*, in (*a–c*) left forelimb, (*d–f*) right forelimb, both shown as mounted in (*a*)*,* (*d*) medial, (*b,e*) lateral and (*c,f*) anterior views. dpc, deltopectoral crest; hu, humerus; mc, metacarpals; ra, radius; sc, scapula; scac, acromial ridge; scb, scapula blade; scgf, glenoid fossa; scgp, glenoid process; st, sternum; ul, ulna. The greyed out area indicates reconstruction. Scale bar, 5 cm.

The scapula is described with the long axis of the blade oriented horizontally, as it is mounted (figure 8, sc). The left scapula is complete, except for the dorsal-most part of the acromial process and proximal plate, and a small portion of the dorsal margin of the distal blade (figure 8*a*,*b*). However, it has been crushed transversely and the blade is distorted. The right scapula lacks the distal end of the blade (figure 8*d*,*e*). Measurements are provided in table 2.

**Table 2.** Measurements of appendicular elements of NHMUK PV R 39000, *Enigmacursor mollyborthwickae*.

element	dimension	value (mm)
right scapula	maximum dorsoventral height of the proximal plate	40.7
right scapula	minimum dorsoventral height of the blade	14.7
left scapula	anteroposterior length	113
left scapula	maximum dorsoventral width of the distal end of the blade	46.8
left scapula	minimum dorsoventral height of the blade	18.7
right sternum	maximum dorsoventral height	40.9
right sternum	maximum transverse width	21.7
left humerus	length	99.5
left humerus	maximum width of the proximal expansion	23.7
left humerus	maximum width of the distal expansion	19.6
left humerus	minimum shaft diameter	11.1
right humerus	length	100.5
right humerus	maximum width of the proximal expansion	24.8 (as preserved)
right humerus	maximum width of the distal expansion	19.6
right humerus	minimum shaft diameter	10.2
left ulna	length	77.4
left ulna	maximum width of the proximal expansion	19.5
left ulna	maximum width of the distal expansion	14.3
left ulna	minimum shaft diameter	8.3
light ulna	length	78.2 (as preserved)
right ulna	maximum width of the proximal expansion	19.4
right ulna	maximum width of the distal expansion	13.1 (as preserved)
right ulna	minimum shaft diameter	7.4
left radius	total length	81.3
left radius	maximum transverse width of the distal expansion	9.0
left radius	minimum shaft diameter	5.9
right radius	maximum transverse width of the distal expansion	11.0
right radius	minimum shaft diameter	5.4
left metacarpal II	total length	14.0
left metacarpal II	minimum shaft diameter	5.6
left metacarpal III	total length	20.2
left metacarpal III	minimum shaft diameter	5.0
left metacarpal IV	total length	18.9
left metacarpal IV	minimum shaft diameter	3.4
left ilium	total length (as preserved)	135.3
left ilium	postacetabular length (as preserved)	44.6
left ilium	height of ilium above the acetabulum	34.7
right ischium	height of the proximal plate	30.6
right femur	total length	169
right femur	minimum shaft circumference	65
right femur	minimum shaft mediolateral width	17.7
right femur	minimum shaft anteroposterior width	20
right femur	maximum mediolateral width of the proximal end	35.5
right femur	maximum anteroposterior length of the greater trochanter	26.9
right femur	distance from the top of the femur to the base of the fourth trochanter	79
right femur	mediolateral width of the distal end	28.3
right femur	maximum anteroposterior width of the distal end	35.4
right tibia	total length	209
right tibia	maximum mediolateral (= anteroposterior) width of the proximal end	40.3
right tibia	maximum anteroposterior (= mediolateral) width of the proximal end	29.2
right tibia	maximum transverse width of the distal end	36.5
right tibia	maximum width of the medial malleolus	19.3
right tibia	minimum shaft diameter	14.3
right tibia	minimum shaft circumference	49
left tibia	minimum shaft diameter	19.1
left tibia	maximum transverse width of the distal end	38.6
left tibia	maximum anteroposterior width of the distal end	18.1
left tibia	minimum shaft circumference	60
left fibula	total length	197
left fibula	maximum anteroposterior width of the proximal end	28.5
left fibula	maximum mediolateral width of the proximal end	9.3
left fibula	maximum anteroposterior width of the distal end	14.1
left fibula	maximum mediolateral width of the distal end	12.2
right astragalus	maximum mediolateral width	30.5
right astragalus	maximum anteroposterior width	23.2
left metatarsal 1	mediolateral width of the distal end	11.8
right metatarsal 1	mediolateral width of the distal end	11.6
left metatarsal 2	total length	91.6
left metatarsal 2	maximum mediolateral width of the proximal end	8.6
left metatarsal 2	maximum anteroposterior width of the proximal end	25.5
left metatarsal 2	maximum mediolateral width of the distal end	14.4
left metatarsal 2	maximum anteroposterior width of the distal end	15.1
right metatarsal 2	total length	96.8
right metatarsal 2	maximum mediolateral width of the proximal end	11.2
right metatarsal 2	maximum anteroposterior width of the proximal end	21.5
right metatarsal 2	maximum mediolateral width of the distal end	14.0
right metatarsal 2	maximum anteroposterior width of the distal end	15.4
left metatarsal 3	maximum mediolateral width of the distal end	18.0
left metatarsal 3	maximum anteroposterior width of the distal end	14.5
right metatarsal 3	maximum mediolateral width of the distal end	18.9
right metatarsal 3	maximum anteroposterior width of the distal end	14.0
left metatarsal 4	maximum mediolateral width of the distal end	14.2
left metatarsal 4	maximum anteroposterior width of the distal end	18.8
right metatarsal 4	total length	88.1
right metatarsal 4	maximum mediolateral width of the proximal end	16.9
right metatarsal 4	maximum anteroposterior width of the proximal end	14.3
right metatarsal 4	maximum mediolateral width of the distal end	15.0
right metatarsal 4	maximum anteroposterior width of the distal end	19.5

In lateral view (figure 8*b*,*e*), the proximal plate is gently concave dorsoventrally (figure 8*b*,*e*, scpp). A well-developed acromial ridge (figure 8*e*, scac) extends from level with the midpoint of the base of the scapula blade, trending anterodorsally to form the anterodorsal corner of the proximal plate. The posterodorsal margin of the proximal plate trends strongly anterodorsally to a level well beyond the dorsal margin of the scapula blade. The dorsoventral projection of the acromial ridge beyond the dorsal margin of the scapula blade is similar to that of *Hexinlusaurus multidens* [27], *Agilisaurus louderbacki* [34], *Hypsilophodon foxii* [31], *Haya griva* [30], *Camptosaurus aphanoecetes* [40] and *Mahuidacursor lipanglef* [44], but much greater than that in *Parksosaurus warreni* [52], *Iani smithi* [41] and *Tenontosaurus tilletti* [43]. The posteroventral margin of the proximal plate is strongly concave in lateral view, forming the posterior margin of the triangular glenoid process (figure 8*e*, scgp). The glenoid fossa (figure 8*e*, scgf) is shallowly concave anteroposteriorly and its articular surface is oriented anterolaterally. A supraglenoid fossa, a feature observed in several early diverging iguanodontians including *Dryosaurus elderae* (CM 11 340), *Cumnoria prestwichii* [47], *Camptosaurus* sp. [40] and *Dysalotosaurus lettowvorbecki* (MB R.1707), is absent. The articular surface for the coracoid is rugose and the two bones were clearly unfused. Medially, the surfaces of both proximal plates are obscured by armature (figure 8*a*,*d*).

The scapula blade (figure 8*a*, scb) has a dorsoventrally narrow base and a strongly flared distal end. It has a straight dorsal margin and a concave ventral margin in lateral view and its lateral surface is slightly convex dorsoventrally. The asymmetrical flaring of the distal end is very similar to that in *Hexinlusaurus multidens* [27], *Agilisaurus louderbacki* [34], *Minimocursor phunoiensis* [38], *Changchunsaurus parvus* [51], *Orodromeus makelai* [36], *Hypsilophodon foxii* [31], *Haya griva* [30] and *Iani smithi* [41], but differs from the more subtly flared distal scapula blades seen in *Eocursor parvus* [26], *Parksosaurus warreni* [52] and *Cumnoria prestwichii* [47]. In the left scapula, what is preserved of the posterior margin is gently convex dorsoventrally. In medial view, the scapula blade is flat, with the exception of a short ridge that extends from the proximal plate for approximately one-third of the length of the blade, after which it merges into the blade’s medial surface. This ridge is situated at a point approximately one-third of the distance from the ventral margin of the blade. In dorsal view, the right scapula blade is bowed laterally.

### 4.9. Right sternal

An elliptical element, with its longest axis trending dorsoventrally, might represent the right sternal (figure 8, st). It is approximately twice as long dorsoventrally as it is wide transversely. Its anterior surface is dorsoventrally and mediolaterally convex, while its posterior surface is flat. Its medial and dorsolateral margins are slightly rugose, whereas an elongated facet is present ventrolaterally that is anteroposteriorly concave and might represent the articular region for the sternal ribs. The sterna of most early diverging neornithischians and ornithopods are reniform (e.g. *Haya griva*, *Changchunsaurus parvus, Hypsilophodon foxii, Tenontosaurus tilletti, Parksosaurus warreni* [30,31,43,51,52]), but it is likely that the sternal is incomplete in *Enigmacursor*, and the true shape is unknown. Measurements are provided in table 2.

### 4.10. Humerus

Both humeri are well preserved, lacking only the margins of the proximal expansion in the right humerus (figure 8, hu). The distal expansions of both humeri are slightly crushed anteroposteriorly. Measurements are provided in table 2.

In anterior view, the proximal end of the humerus is expanded transversely with respect to the shaft (figure 8*c*). The dorsal margin of the proximal end is upwardly convex, and lacks clearly developed lateral or medial tuberosities and there is no clear demarcation between the humeral head and the rest of the proximal margin, as also occurs in *Lesothosaurus diagnosticus* [25], *Eocursor parvus* [26], *Hexinlusaurus multidens* [27], *Orodromeus makelai* [36], *Sanxiasaurus modaoxiensis* [39] and *Hypsilophodon foxii* [31]. In posterior view, a poorly defined humeral head is present. It is rounded in outline and projects slightly posteriorly to overhang the posterior surface.

In anterior view, the proximal expansion is medially inclined, with its lateral margin forming an angle of approximately 20° with respect to the shaft, as also seen in *Eocursor parvus* [26], *Hexinlusaurus multidens, Yandusaurus hongheensis* [27], *Orodromeus makelai* [36], *Sanxiasaurus modaxoiensis* [39] and *Hypsilophodon foxii* [31], but differing from *Lesothosaurus diagnosticus* [25] in which it is parallel with the shaft. The anterior surface of the proximal expansion is flat to very slightly concave transversely. By contrast, the posterior surface is transversely convex. In lateral view, the humerus is straight and lacks significant curvature (figure 8*b*,*e*), as in *Lesothosaurus diagnosticus* [25], *Eocursor parvus* [26], *Sanxiasaurus modaoxiensis* [39] and *Hexinlusaurus multidens* [27]. This contrasts with the condition in *Orodromeus makelai* [36] and *Hypsilophodon foxii* [31], where the humerus is sigmoidal in lateral view.

Further ventrally, in anterior view, the deltopectoral crest (figure 8, dpc) is a low, triangular projection that is situated approximately one-third of the way down the shaft, on its lateral margin, and its apex is thickened slightly transversely, similar to *Lesothosaurus diagnosticus* [25]. In contrast, the apex of the deltopectoral crest appears to be more proximally situated in *Jeholosaurus shangyuanensis* [29], while it is more distally situated in *Tenontosaurus tilletti* [43] and *Camptosaurus* [40]. In lateral view, the crest projects anteriorly and its dorsal margin is slightly concave, as in *Hexinlusaurus multidens* [27], *Orodromeus makelai* [36], *Haya griva* [30] and *Hypsilophodon foxii* [31]. The deltopectoral crest is relatively small in *Enigmacursor,* whereas it is better developed in *Eocursor parvus* [26], *Hypsilophodon foxii* [31] and especially *Tenontosaurus tilletti* [43] and *Camptosaurus* [40]. The ventral margin of the deltopectoral crest of *Enigmacursor* is straight and merges gently with the shaft. A faint intermuscular line extends ventrally from a point approximately halfway along the dorsal margin of the deltopectoral crest, fading just ventral to the apex of the crest. Anterior to this intermuscular line, the lateral surface of the deltopectoral crest is slightly rugose, as in *Hypsilophodon foxii* [31]. Ventral to the deltopectoral crest, the lateral and medial margins of the humerus converge to form the shaft. The shaft margins extend parallel to each other before flaring transversely to form the distal expansion, which is less strongly expanded than the proximal end. The anterior surface of the shaft is flat to slightly convex, whereas its posterior surface is more strongly convex, giving it a ‘D’-shaped cross section.

In anterior view, the distal expansion is slightly asymmetrical with a slightly convex medial margin and shallowly concave lateral border. It extends slightly more medially, with respect to the shaft, than laterally, as also seen in *Lesothosaurus diagnosticus* [25] and *Eocursor parvus* [26]. A shallow fossa extends over the anterior surface of the distal expansion. Posteriorly, the surface is slightly crushed, but there is no clear olecranon fossa. This appears to differ from the condition in *Hexinlusaurus multidens* [27] and *Tenontosaurus tilletti* [43], where a clear olecranon fossa is present posteriorly. In anterior or posterior view, the ventral margin of the humerus is straight. In distal view, the condyles are slightly crushed anteroposteriorly, giving a lenticular outline, but it seems likely that the medial and lateral condyles were originally sub-equal in both length and width and separated from each other by a shallow midline groove, as in *Hexinlusaurus multidens* [27].

### 4.11. Ulna

The left ulna is complete while the right is missing its proximal-most and distal-most ends (figure 8, ul). The proximal end of the left ulna is crushed anteroposteriorly. Measurements are provided in table 2.

In anterior or lateral view, the ulna is straight, unbowed and slender (figure 8, ul). By contrast, the ulna of *Iani smithi* [41] is bowed in both medial and anterior views, while that of *Tenontosaurus tilletti* [43] is bowed in medial view but straight in anterior view. In *Enigmacursor*, *the* original shape of the proximal end has been altered by crushing; however, it appears to have been more strongly expanded transversely than either the shaft or distal end. In anterior view, the proximal end is almost symmetrical with respect to the long axis of the bone. A low, rounded olecranon process is present, and projects dorsally, but is weakly offset from the rest of the proximal expansion and is slightly expanded anteroposteriorly with respect to the rest of the proximal end, as in *Lesothosaurus diagnosticus* [25] and *Hexinlusaurus multidens* [27]. The olecranon process appears to be better developed in *Haya griva* [30], *Parksosaurus warreni* [52], *Orodromeus makelai* [36], *Hypsilophodon foxii* [31], *Iani smithi* [41] and iguanodontians such as *Cumnoria prestwichii* [47], *Tenontosaurus tilletti* [43] and *Camptosaurus* spp [40]. The poorly defined medial process has a triangular outline in anterior view and is anteroposteriorly compressed. In proximal view, the ulna has an oval outline that tapers medially, and lacks the anterior process present in iguanodontians such as *Tenontosaurus tilletti, Cumnoria prestwichii* and *Camptosaurus dispar* [40,43,47].

Ventral to the proximal expansion, the shaft has an elliptical transverse cross section and is otherwise featureless, lacking clear muscle scars or intermuscular lines, in contrast to *Haya griva*, in which a ridge extends across the shaft [30]. Ventrally, the shaft expands slightly transversely and anteroposteriorly to form the distal expansion. The anterior surface of the distal end is transversely concave, presumably to receive the distal end of the radius, whereas the posterior surface is gently convex. This gives the distal end of the ulna a ‘U’-shaped cross section. This continues ventrally so that in distal view, a shallow depression invaginates its anterior margin, as in *Orodromeus makelai* [36]. The distal articular surface of the ulna is smoothly convex anteroposteriorly.

### 4.12. Radius

The left radius is complete, although its proximal end is crushed and its posterior surface eroded (figure 8, ra). The distal end of the right radius is reconstructed. Measurements are provided in table 2.

In anterior and lateral views, the radius is straight, elongated and slender, and lacks significant curvature in any direction (figure 8, ra), as in *Lesothosaurus diagnosticus* [25] and most ornithopods including *Hypsilophodon foxii* [31] and *Haya griva* [30]. In proximal view, the radius is anteroposteriorly flattened, with an elongated elliptical outline. The proximal and distal ends are slightly expanded transversely relative to the shaft and expand to roughly the same degree. This differs from the condition in *Dryosaurus elderae* (CM 11340), *Cumnoria prestwichii* [47] and *Tenontosaurus tilletti* [43], where the distal end is more expanded than the proximal end, and *Haya griva* [30] and *Parksosaurus warreni* [52] where the proximal end is more expanded than the distal end. The anterior surface of the radius is convex along its entire length, whereas its posterior surface is convex in its proximal part but flattens distally, forming an articular surface that would have articulated with the ulna. Consequently, the distal end has a ‘D’-shaped cross section. The distal articular surface is anteroposteriorly convex.

### 4.13. Manus

As mounted, the right manus contains no original bone material. By contrast, three metacarpals are included in the left manus and are mounted as metacarpals II–IV (figure 8*a*–*c*, mc). All three metacarpals are poorly preserved and have a simple, dumbbell-shaped morphology. All are longer proximodistally than they are wide transversely, with a central shaft separating slightly expanded proximal and distal articular surfaces. As mounted, metacarpal III is the longest; metacarpal IV is slightly shorter than metacarpal III, but more gracile; and metacarpal II is the shortest but proportionally widest of the three. It is possible that metacarpals II and IV are mounted the wrong way around, because in *Orodromeus makelai* [36], *Hypsilophodon foxii* [31], *Hexinlusaurus multidens* [27], *Minimocursor phunoiensis* [38] and *Tenontosaurus tilletti* [43], metacarpal II is slightly shorter than metacarpal III, with metacarpal IV being the shortest of the three. However, in those taxa, metacarpal IV is also the most slender, and in *Lesothosaurus diagnosticus* [25] metacarpal II is shorter than metacarpal IV. In *Changmiania liaoningensis*, metacarpal III is apparently the shortest [49]. The proximal and distal articular surfaces are largely obscured by the reconstructed carpus and phalanges. Collateral ligament pits are present on the distal ginglymi of metacarpal II but cannot be seen on metacarpals III and IV. The shafts of metacarpals II and III are sub-quadrate in cross section, whereas that of metacarpal IV is rounded. Measurements are provided in table 2.

### 4.14. Ilium

**Figure 9.**
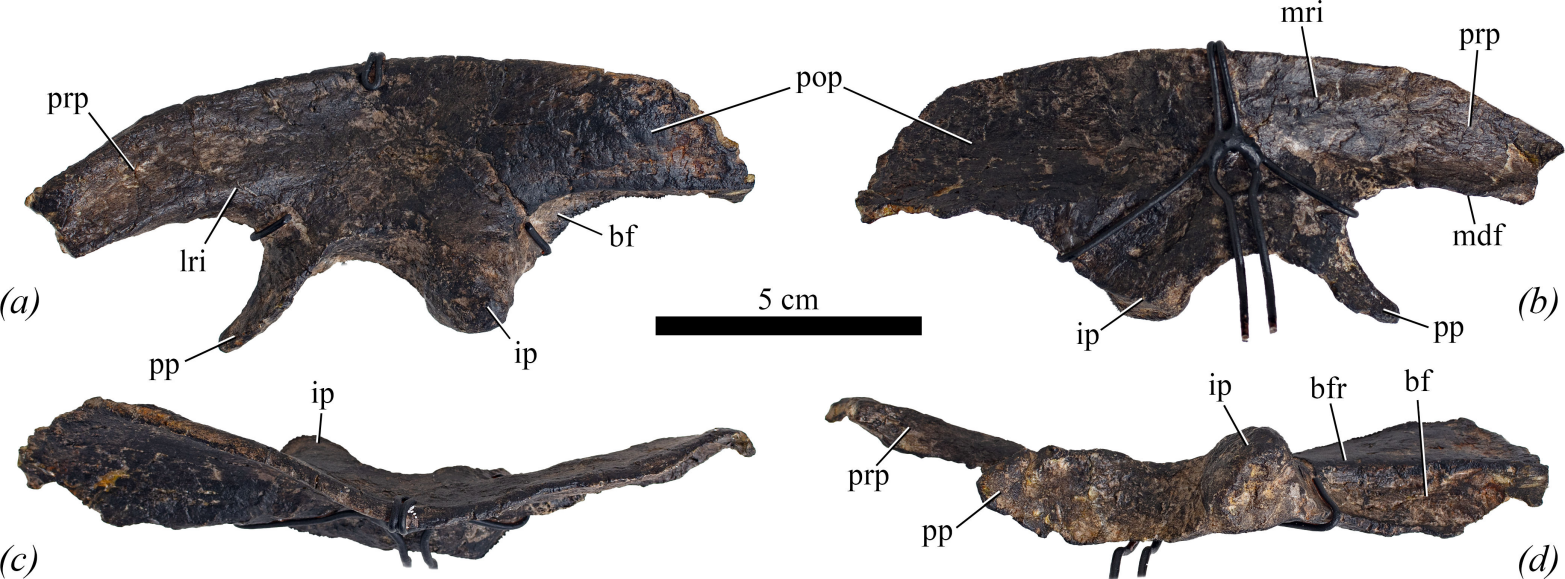
Left ilium of NHMUK PV R 39000, *Enigmacursor mollyborthwickae*, in (*a*) lateral, (*b*) medial, (*c*) dorsal and (*d*) ventral views. bf, brevis fossa; bfr, ridge bounding the brevis fossa laterally; ip, ischiadic peduncle; lri, ridge on the lateral surface extending posteriorly from the preacetabular process; mdf, medially directed flange on the ventral margin of the preacetabular process; mri, ridge on the medial surface, extending forward to the preacetabular process; pp, pubic peduncle; pop, postacetabular process; prp, preacetabular process. Scale bar, 5 cm.

The left ilium is largely complete, missing only the anterior-most tip of the preacetabular process and the posterior margin of the postacetabular process (figure 9), while the right ilium is entirely reconstructed. Measurements are provided in table 2.

In dorsal view, the ilium describes a sigmoidal curve, with the preacetabular process extending anteriorly and slightly laterally, the region above the acetabulum extending medially and the postacetabular process extending posteriorly and slightly laterally, except in its posterior-most part, which curves posteromedially (figure 9*c*). In lateral view, the ilium is elongated and low and has a gently convex dorsal margin lacking distinct breaks in slope (figure 8*a*), similar to early diverging ornithischians (e.g. *Lesothosaurus diagnosticus* [25]) and neornithischians (e.g. *Haya griva* [30]) but differing from iguanodontians such as *Valdosaurus canaliculatus* [54], *Tenontosaurus tilletti* [43] and *Camptosaurus* sp. [40], which have iliac dorsal margins that are sinuous in lateral view to varying degrees. The preacetabular process of the ilium in *Enigmacursor* has sub-parallel dorsal and ventral margins and tapers only slightly as it extends anteriorly (figure 9*a*, prp). It is transversely compressed along its length, as in *Lesothosaurus diagnosticus* [25], *Jeholosaurus shangyuanensis* [29] and *Haya griva* [30] but differs from those of *Dryosaurus elderae* (CM 3392) and *Camptosaurus* sp. [40] in which the preacetabular process rotates as it extends anteriorly so that it is anteroposteriorly compressed. Although broken, it is clear that the preacetabular process of *Enigmacursor* extended considerably anterior to the pubic peduncle (figure 9*a*). Its lateral surface is flat to very gently concave dorsoventrally. Its ventral margin merges smoothly into the anterior surface of the pubic peduncle forming a broad, ‘U’-shaped trough. In medial view (figure 9*b*), the preacetabular process is dorsoventrally concave, forming an anteroposteriorly trending groove that presumably accommodated several sacral ribs. This concavity is due to the presence of a medially directed flange that forms the ventral margin of the process, giving it an inverted ‘L’-shaped transverse cross section, as also seen in *Hexinlusaurus multidens* [27], *Jeholosaurus shangyuanensis* [29] and *Hypsilophodon foxii* [31].

The main body of the ilium is anteroposteriorly and dorsoventrally concave in lateral view (figure 9*a*). There is no distinct supraacetabular crest above the acetabulum, in contrast to *Lesothosaurus diagnosticus* [25], *Eocursor parvus* [26] and *Minimocursor phunoiensis* [38], but similar to *Hexinlusaurus multidens* [27], *Jeholosaurus shangyuanensis* [29], *Haya griva* [30] and most neornithischians [28]. A subtle ridge extends from the ventral margin of the preacetabular process posteriorly, fading after a short distance (figure 9*a*, lri); this ridge is absent in *Dryosaurus elderae* (CM 3392, CM 21786). In medial view, a distinct ridge arises at a point just ventral to the dorsal margin of the ilium level with the midpoint of the acetabulum and extends anteriorly until it merges with the dorsal margin of the preacetabular process just anterior to the pubic peduncle (figure 9*b*, mri). This ridge forms the dorsal margin of the same trough that is present on the medial margin of the preacetabular process. Ventrally, this trough is defined by a swollen area immediately dorsal to the acetabulum. There are no distinct individual sacral rib facets within this trough. In comparison to the dorsal part of the iliac blade, which is transversely compressed, the acetabular region is transversely expanded.

The pubic peduncle (figure 9, pp) is a finger-like process that is much longer dorsoventrally than anteroposteriorly. In lateral view, the process curves anteroventrally towards its tip and terminates in a blunt rounded apex. The base of the pubic peduncle has a sub-triangular transverse cross section with the apex of this triangle forming the anterodorsal margin of the process. The medial and lateral surfaces of the peduncle are flat to gently concave, whereas the posterior surface is flat and forms the anterior-most part of the acetabular margin (figure 9*d*, pp). The acetabulum is fully open and lacks a ventromedial flange, similar to the condition in *Eocursor parvus* [26], *Hexinlusaurus multidens* [27] and neornithischians [28], but differing from *Lesothosaurus diagnosticus* [25] and *Agilisaurus louderbacki* [26], which both possess a ventromedial flange. The ischiadic peduncle (figure 9, ip) is much longer than the pubic peduncle anteroposteriorly, and forms a stout triangular process in lateral view with the apex pointing ventrally (figure 9*a*, ip). It has a semicircular transverse cross section with a straight anterior margin and continuous lateral, posterior and medial margins that merge into each other without a distinct break-in-slope. The anterior surface of the ischiadic peduncle is oriented slightly laterally with respect to the rest of the acetabulum (figure 9*c*,*d*, ip) so that in ventral view the posterior part of the acetabular margin is slightly kinked relative to its middle and anterior part.

The postacetabular process (figure 9, pop) is longer anteroposteriorly than it is tall dorsoventrally in lateral view and its lateral surface is gently convex both dorsoventrally and anteroposteriorly (figure 9*a*). There is a prominent brevis fossa (figure 9, bf) whose anterior part is visible in lateral view, as in *Lesothosaurus diagnosticus* [25], *Minimocursor phunoiensis* [38], *Haya griva* [30], *Hypsilophodon foxii* [31], *Parksosaurus warreni* [52] and *Thescelosaurus neglectus* [46]. This contrasts with the condition in *Jeholosaurus shangyuanensis* [29], *Changmiania liaoningensis* [49], *Valdosaurus canaliculatus* [54] and *Dryosaurus elderae* (CM 3392) in which the brevis fossa is not visible in lateral view. The ventral margin of the postacetabular process merges with the posterior margin of the ischiadic peduncle to form a ridge that defines the lateral margin of the brevis fossa (figure 9*d*, bfr). In ventral view, the brevis fossa is shallowly concave anteroposteriorly and strongly concave transversely (figure 9*d*). The brevis fossa is transversely narrowest anteriorly and widens slightly as it expands posteriorly, as in *Eocursor parvus* [26] and *Orodromeus makelai* [36], but in contrast to *Hypsilophodon foxii* [31] and *Parksosaurus warreni* [52] where it is parallel-sided, and *Valdosaurus canaliculatus* [54] where it flares significantly more. Although the postacetabular process is broken posteriorly, only a small amount appears to be missing, so it is unlikely that it would have been further expanded than the preserved portion. In medial view, the postacetabular process is strongly concave dorsoventrally due, in part, to the presence of the horizontal brevis shelf ventrally. As with the rest of the ilium, it is transversely compressed, and when viewed posteriorly, has a ‘C’-shaped cross section (figure 9*b*, pop).

### 4.15. Pubis

**Figure 10.**
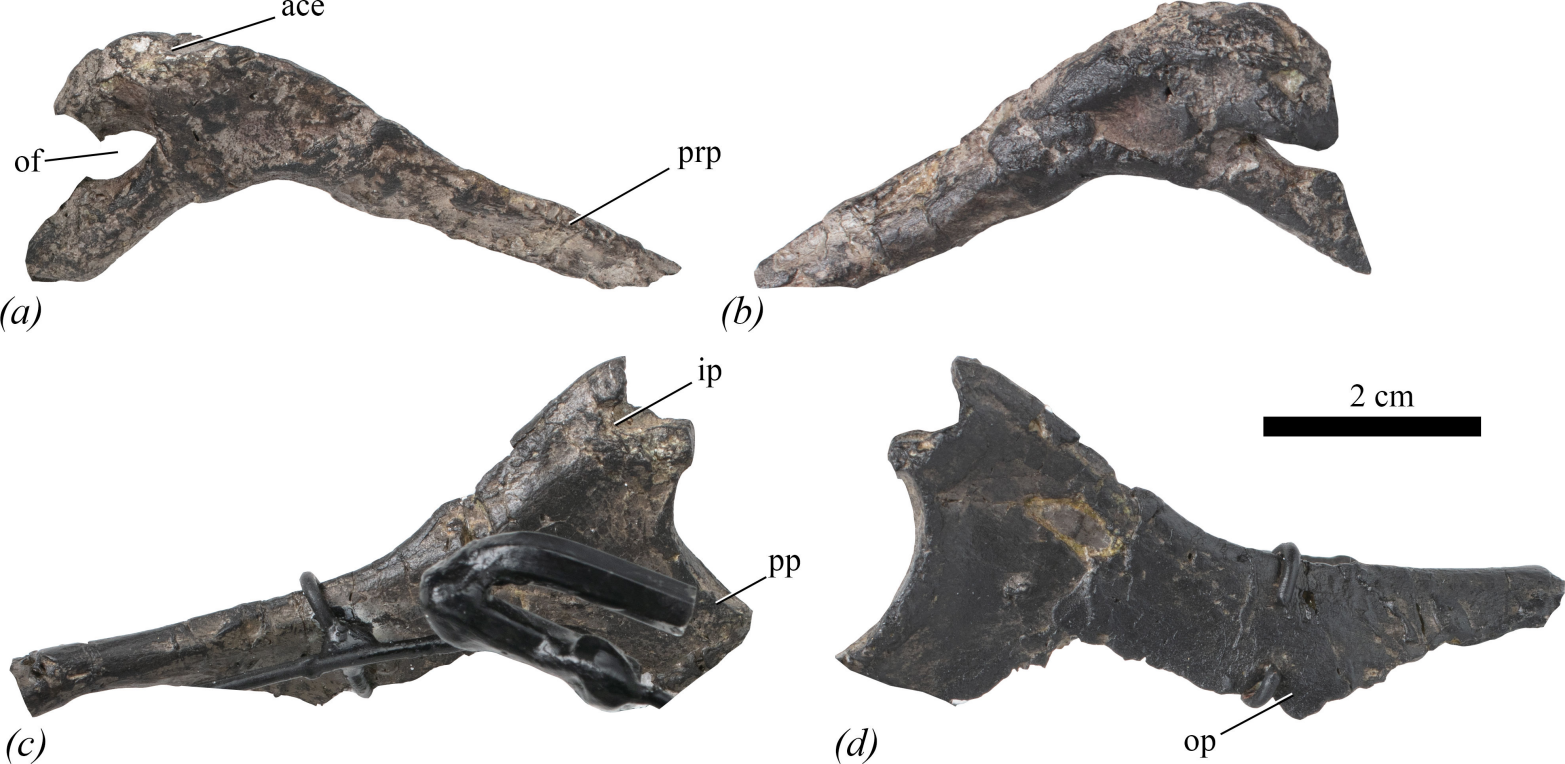
Ischium and pubis of NHMUK PV R 39000, *Enigmacursor mollyborthwickae*, in (*a*,*b*) right pubis, (*c*,*d*) right ischium in (*a*)*,* (*c*) lateral and (*b*,*d*) medial views. The ischium and pubis are mounted along with the reconstructed left elements, and these and the reconstructed parts of the right elements have been cropped from the photographs for clarity; however, metalwork still obscures the lateral surface of the right ischium. ace, acetabular region of the pubis; ip, iliac peduncle of the ischium; of, obturator foramen; op, obturator process; pp, pubic peduncle of the ischium; prp, prepubis. Scale bar, 2 cm.

The right pubis is represented by the prepubic process (with the exception of its anterior-most tip, which is reconstructed) and a section of its acetabular region only; the region behind the obturator foramen and the postpubic process are entirely reconstructed (figure 10*a*,*b*). The left pubis is not preserved. On the mounted skeleton, the right pubis has been mounted as the left. Measurements are provided in table 2.

In lateral view, the preserved part of the pubis is boomerang-shaped with the prepubis projecting anteriorly and ventrally (figure 10*a*). The prepubic process is transversely compressed with sharp dorsal and ventral margins (figure 10*a*,*b*, pp), as in *Jeholosaurus shangyuanensis* [29], but in contrast to *Hypsilophodon foxii* (NHMUK PV R 193), in which the prepubis is slightly broader transversely than it is deep dorsoventrally. The medial surface of the prepubis is slightly dorsoventrally convex while its lateral surface is flat. The dorsal and ventral margins of the prepubis are straight and converge anteriorly. The prepubis, although not complete, appears to be longer than it is in *Hexinlusaurus multidens* [27]. The acetabular region of the pubis (figure 10*a*,*b*, ace) is transversely expanded relative to the prepubis and forms the apex of the ‘boomerang’. Its lateral surface exhibits signs of crushing but was likely gently convex dorsoventrally, whereas its medial surface is flat (figure 10*b*), lacking the boss for articulation of a sacral rib observed in *Orodromeus makelai* [36] and *Haya griva* [30]. Due to poor preservation, the dorsal surface of the main body lacks a distinct division between the region for the articulation of the pubic peduncle of the ilium and the acetabular margin; however, this entire area is roughened and rugose. The posterior margin of the acetabular region is angled posteroventrally and then curves anteriorly to form the dorsal margin of the obturator foramen (figure 10*a*, of). The obturator foramen appears to be smaller relative to the acetabular region than in *Lesothosaurus diagnosticus* [25] and *Eocursor parvus* [26]. The ventral margin of the obturator foramen is formed by a rod-like process that extends posteroventrally and would have formed the proximal part of the postpubic rod. This rod-like process has an ellipsoidal transverse cross section, as *Haya griva* [30], *Orodromeus makelai* [36] and *Jeholosaurus shangyuanensis* [29]. It is not clear if the borders of the obturator foramen were originally partially open or closed because this area has been damaged and obscured by reconstruction.

### 4.16. Ischium

The proximal plate of the right ischium and the proximal part of the shaft are preserved (figure 10*c*,*d*) but the shaft distal to the obturator process and the entire left ischium are reconstructed. As with the pubis, the right ischium has been mounted as the left. Measurements are provided in table 2.

In lateral view, the preserved portion of the ischium is roughly ‘Y’-shaped, with an anterodorsally projecting iliac process (figure 10, ip), an anteroventrally projecting pubic process (figure 10, pp) and a posteriorly and slightly ventrally projecting shaft. The proximal plate is transversely compressed except for the iliac process, which is slightly expanded laterally.

In lateral view (figure 10*c*), both the pubic and iliac processes have square outlines and are approximately similar in their dimensions. By contrast, in *Haya griva* [30] and *Jeholosaurus shangyuanensis* [29], the pubic process is longer than the iliac process. The pubic process has a straight anterior margin that is in contact with the pubis. It has a triangular transverse cross section that is broadest dorsally and thins ventrally. Posterior to this, the anterodorsal margin of the proximal plate describes a gentle curve that forms the acetabular margin and is continuous with the anteroventral margin of the iliac process. The articular surface of the iliac process is sub-ellipsoidal in outline and is deeply excavated (although this feature is likely due to erosion).

The shaft extends posteroventrally from the proximal plate. Its proximal region is transversely compressed and dorsoventrally deep, with parallel dorsal and ventral margins. The dorsal margin of the shaft curves gently posteriorly, while the ventral margin is inflected to form the base of a triangular obturator process (figure 10, op), but this region has been broken, so the original shape of the complete process is unknown. Although the shape of the obturator process is unknown, it appears to have been situated in roughly the same location as that of *Hexinlusaurus multidens* [27] and *Jeholosaurus shangyuanensis* [29], whereas those of *Haya griva* [30], *Hypsilophodon foxii* (NHMUK PV R 193 [31]) and *Parksosaurus warreni* [52] are more distally located. The shaft is slightly dorsoventrally concave in the region of the obturator process. Around this inflexion point, the ventral margin of the shaft undergoes torsion to become the medial margin, while concurrently the dorsal margin rotates slightly laterally, as in *Hypsilophodon foxii* (NHMUK PV R 193). At the point where the shaft is broken, it has a teardrop-shaped transverse cross section, with its long axis oriented mediolaterally.

### 4.17. Femur

**Figure 11.**
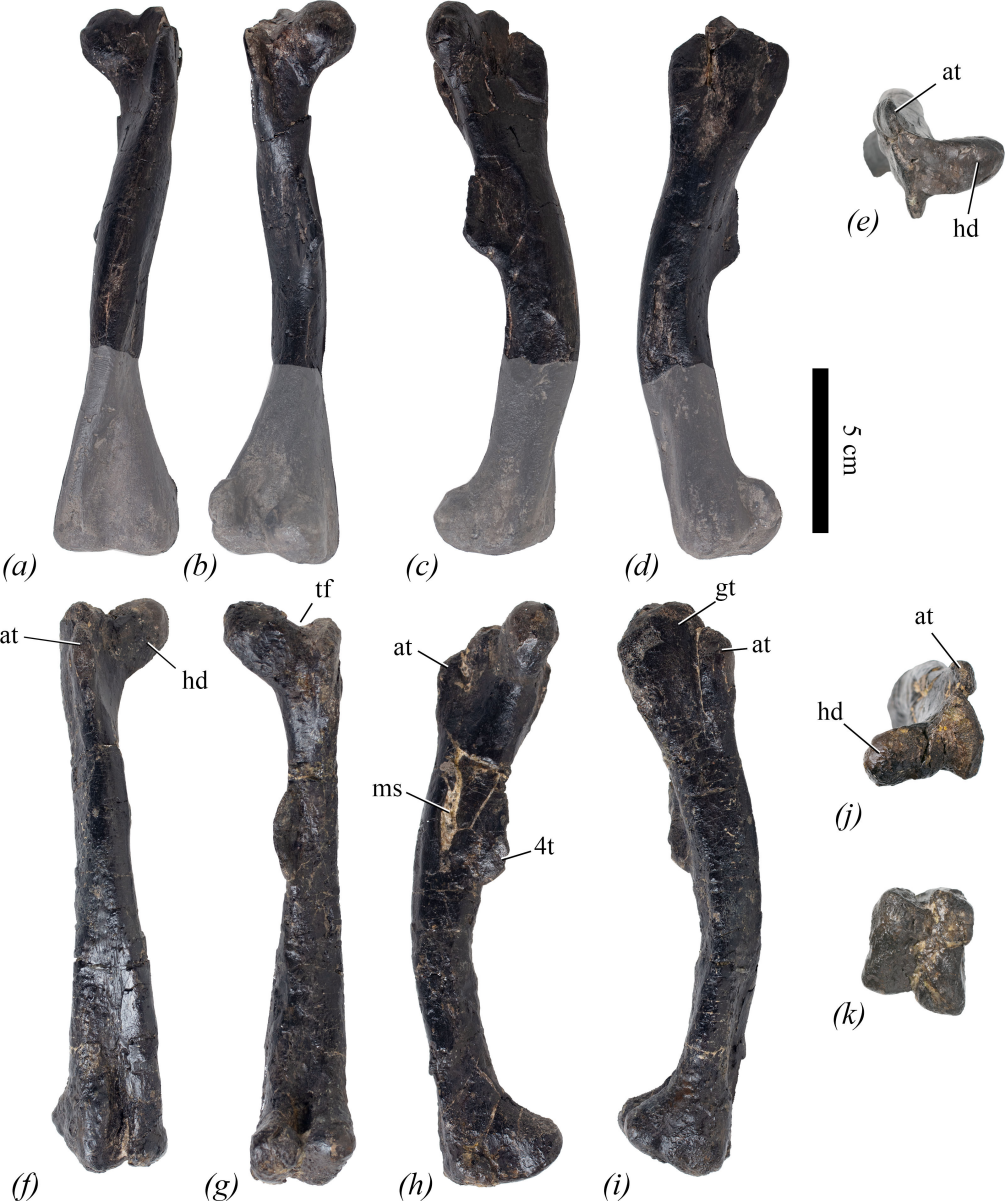
Femora of NHMUK PV R 39000, *Enigmacursor mollyborthwickae*, in (*a–e*) left femur; (*f–k*) right femur in (*a*); (*f*) anterior; (*b,g*) posterior; (*c,h*) medial; (*d,i*) lateral; (*e,j*) proximal; and (*k*) distal views. 4t, fourth trochanter; at, anterior trochanter; gt, greater trochanter; hd, head; ms, muscle scar; tf, trochanteric fossa. The greyed out area indicates reconstruction. Scale bar, 5 cm.

The right femur is complete, although its distal end is slightly crushed (figures 1 and 11). The left femur is missing its distal third and has a heavy coating of plaster across its surface; consequently, the description is primarily based on the right element. Measurements are provided in table 2.

In anterior view, the femur is straight (figure 11*a*,*f*), whereas in medial view it is gently bowed anteriorly (figure 11*c*,*h*), as in most small ornithischians such as *Lesothosaurus diagnosticus* [25], *Eocursor parvus* [26], *Hexinlusaurus multidens* [27], *Agilisaurus louderbacki* [34], *Haya griva* [30] and *Jeholosaurus shangyuanensis* [29]. In anterior view, the head of the femur is orientated medially and slightly dorsally and projects dorsal to the greater trochanter (figures 1a,g,h,11). The head is globose and rounded and is separated from the greater trochanter by a shallow trochanteric fossa (figures 1a,g,11, tf), similar to that in neornithischians such as *Minimocursor phunoiensis* [38], *Haya griva* [30], *Changchunosaurus parvus* [51], *Jeholosaurus shangyuanensis* [29] and *Hypsilophodon foxii* (figure 1*g*) [31], but in contrast to the condition in *Lesothosaurus diagnosticus* (figure 1*d*) [25], *Eocursor parvus* [26], *Sanxiasaurus modaoxiensis* [39] and *Hexinlusaurus multidens* [27], where the head is more transversely compressed and not clearly separated from the greater trochanter. The anterior surface of the head is dorsoventrally convex. Its dorsal margin is straight whereas its dorsomedial margin is curved, and its ventral margin forms an angle of approximately 120° with the medial margin of the shaft. A saddle-shaped sulcus separates the anterior surface of the head from the rest of the proximal end. In posterior view, the surface of the head is convex (figures 1a,g,11), lacking a ligament sulcus. By contrast, a deep, clear and well-developed ligament sulcus is present in *Minimocursor phunoiensis* [38], *Haya griva* [30], *Changchunosaurus parvus* [51], *Jeholosaurus shangyuanensis* [29], *Hypsilophodon foxii* (figure 1*g*) [31], *Dryosaurus* sp. [50], *Dysalotosaurus* (figure 1*j*, MB R.2511) and *Camptosaurus* sp. [40]. However, this feature appears to be absent or poorly developed in early diverging ornithischians that lack a globose head, such as *Lesothosaurus diagnosticus* (figure 1*d*) [25]. In dorsal view, the head has sub-parallel anterior and posterior margins, and its dorsal surface is strongly convex anteroposteriorly and weakly convex mediolaterally.

The greater trochanter is mediolaterally compressed in anterior and posterior views (figure 1*a*). By contrast, in *Hypsilophodon foxii* (figure 1*g*) [31], *Dryosaurus elderae* (CM 21 786) and *Dysalotosaurus lettowvorbecki* (figure 1*j*, MB R.2511), the greater trochanter is transversely expanded in posterior view due to a rugose muscle scar that extends onto the lateral surface of the shaft. In dorsal view, the greater trochanter of *Enigmacursor* forms the anteroposteriorly widest part of the proximal femur and has a gently convex lateral margin. In lateral view, the greater trochanter has a flat to gently convex surface (figures 1b,i,11, gt). The anterior trochanter is separated from the greater trochanter by a narrow cleft that extends for only a short distance in medial view, but a greater distance in lateral view (figures 1b,h,i,11, at). By contrast, the anterior trochanter of *Dryosaurus* sp. [50] is separated from the greater trochanter by a cleft that extends further ventrally in medial view. The apex of the anterior trochanter is situated below the level of the greater trochanter at a level just above the ventral margin of the femoral head (figures 1b,f,h,11, at), as in *Lesothosaurus diagnosticus* (figure 1*e*) [25], *Eocursor parvus* [26], *Sanxiasaurus modaoxiensis* [39] and *Hexinlusaurus multidens* [27]. This contrasts with the condition in other small ornithischians: in *Haya griva* [30], *Minimocursor phunoiensis* [38] and *Jeholosaurus shangyuanensis* [29], the anterior trochanter is lower than the greater trochanter but higher than the ventral margin of the head; in *Hypsilophodon foxii* (figure 1*h*) [31], *Dryosaurus altus* [50] and *Camptosaurus aphanocetes* [40], the anterior trochanter projects to the same level as the greater trochanter and in *Changchunsaurus parvus* [51] and *Dysalotosaurus lettowvorbecki* (figure 1*k*, MB R.2511), the anterior trochanter projects higher than the greater trochanter. In *Enigmacursor*, the anterior trochanter is a finger-like process with a flattened sub-triangular cross section. In lateral view, its transverse width is much less than that of the greater trochanter (figures 1b,i,11), similar to the condition in *Haya griva* [30], *Minimocursor phunoiensis* [38], *Changchunsaurus parvus* [51], *Jeholosaurus shangyuanensis* [29], *Hypsilophodon foxii* (figure 1*h*) [31], *Dryosaurus altus* [50] and *Camptosaurus aphanocetes* [40], but in contrast to *Lesothosaurus diagnosticus* (figure 1*e*) [25], *Eocursor parvus* [26], *Sanxiasaurus modaoxiensis* [39] and *Agilisaurus louderbacki* [34] in which the greater and anterior trochanters are sub-equal in size in lateral view. In anterior view, the ventral margin of the anterior trochanter is continuous with an intramuscular line that extends for a short distance onto the femoral shaft. The proximal part of the shaft has a sub-triangular cross section that is widest anteriorly and tapers posteriorly. More ventrally, ventral to the fourth trochanter, the cross section becomes sub-quadrangular and is mediolaterally wider than it is anteroposteriorly.

The fourth trochanter (figures 1c,h,11 and 4*t*) is positioned on the proximal half of the femoral shaft, as in *Lesothosaurus diagnosticus* [25], *Eocursor parvus* [26], *Hexinlusaurus multidens* [27], *Minimiocursor phunoiensis* [38], *Haya griva* [30], *Jeholosaurus shangyuanensis* [29], *Hypsilophodon foxii* [31] and most small ornithischians. It arises from the posteromedial corner of the shaft. Its medial surface is dorsoventrally convex, whereas its lateral surface is dorsoventrally concave. Its posterior margin describes a gentle ‘C’-shaped curve as it descends ventrally, so that it is crest-like, although the tip is slightly broken. The ventral margin of the fourth trochanter is straight to slightly outwardly convex (figure 1*c*), and this indicates that its distal tip did not curve ventrally (it was not ‘pendant’). This contrasts with the condition in other early diverging ornithischians, neornithischians and ornithopods (figure 1), in which the ventral margin of the fourth trochanter is strongly upwardly concave (e.g. *Lesothosaurus diagnosticus* (figure 1*f*) [25], *Eocursor parvus* [26], *Hexinlusaurus multidens* [27], *Agilisaurus louderbacki* [34], *Sanxiasaurus modaoxiensis* [39], *Haya griva* [30], *Minimocursor phunoiensis* [38], *Jeholosaurus shangyuanensis* [29], *Tenontosaurus tilletti* [43]). In medial view, a prominent, elongated, slit-like muscle scar (figure 11*h*, ms) is present that is situated immediately anterior to the base of the fourth trochanter. It has a prominent anterior margin and the area posterior to this is deeply depressed. A muscle scar is frequently present in this area in other early diverging ornithischians, neornithischians and ornithopods (e.g. *Eocursor parvus* [26], *Hexinlusaurus multidens* [27], *Haya griva* [30], *Dryosaurus altus* [50]) but is not usually slit like. Although this feature appears to be genuine in *Enigmacursor*, it is likely that it has been accentuated by crushing.

In anterior view, the distal end of the femur is slightly expanded mediolaterally with respect to the shaft (figure 11*f*). An anterior intercondylar groove may have been present but this area has been extensively crushed and it is unclear whether this feature is genuine (figure 11*k*). An anterior intercondylar groove is present in many iguanodontians, including *Dryosaurus elderae* (CM 11 340; CM 21786), *Tenontosaurus tilletti* [43] and *Camptosaurus aphanocetes* [40] but is generally absent in earlier diverging taxa (e.g. *Hexinlusaurus multidens, Yandusaurus hongheensis* [27], *Haya griva* [30], *Jeholosaurus shangyuanensis* [29]). Similarly, there is some evidence for a small lip of bone overlapping this groove distally on the medial side, although it is not clear if this feature is genuine, and it is absent laterally. The posterior surface of the distal end supports two large epicondyles that are divided by a short posterior intercondylar groove (figure 11*g*). This groove is infilled with sediment and crushed and thus it is difficult to discern its original shape. The medial and lateral epicondyles are sub-equal in mediolateral width (figure 11*k*), as in *Eocursor parvus* [26] and *Jeholosaurus shangyuanensis* [29]; in *Haya griva* [30], *Minimocursor phunoiensis* [38], *Dryosaurus elderae* (CM 11340), *Tenontosaurus tilletti* [43] and *Camptosaurus aphanocetes* [40], the medial condyle is mediolaterally narrower than the lateral condyle. In *Enigmacursor,* the medial condyle projects further posteriorly than the lateral condyle in lateral view, as in *Eocursor parvus* [26], *Agilisaurus louderbacki* [34], *Hypsilophodon foxii* [31] and *Dryosaurus elderae* (CM 11340), but in contrast to *Haya griva* [30], where both condyles project posteriorly to the same extent.

### 4.18. Tibia

**Figure 12.**
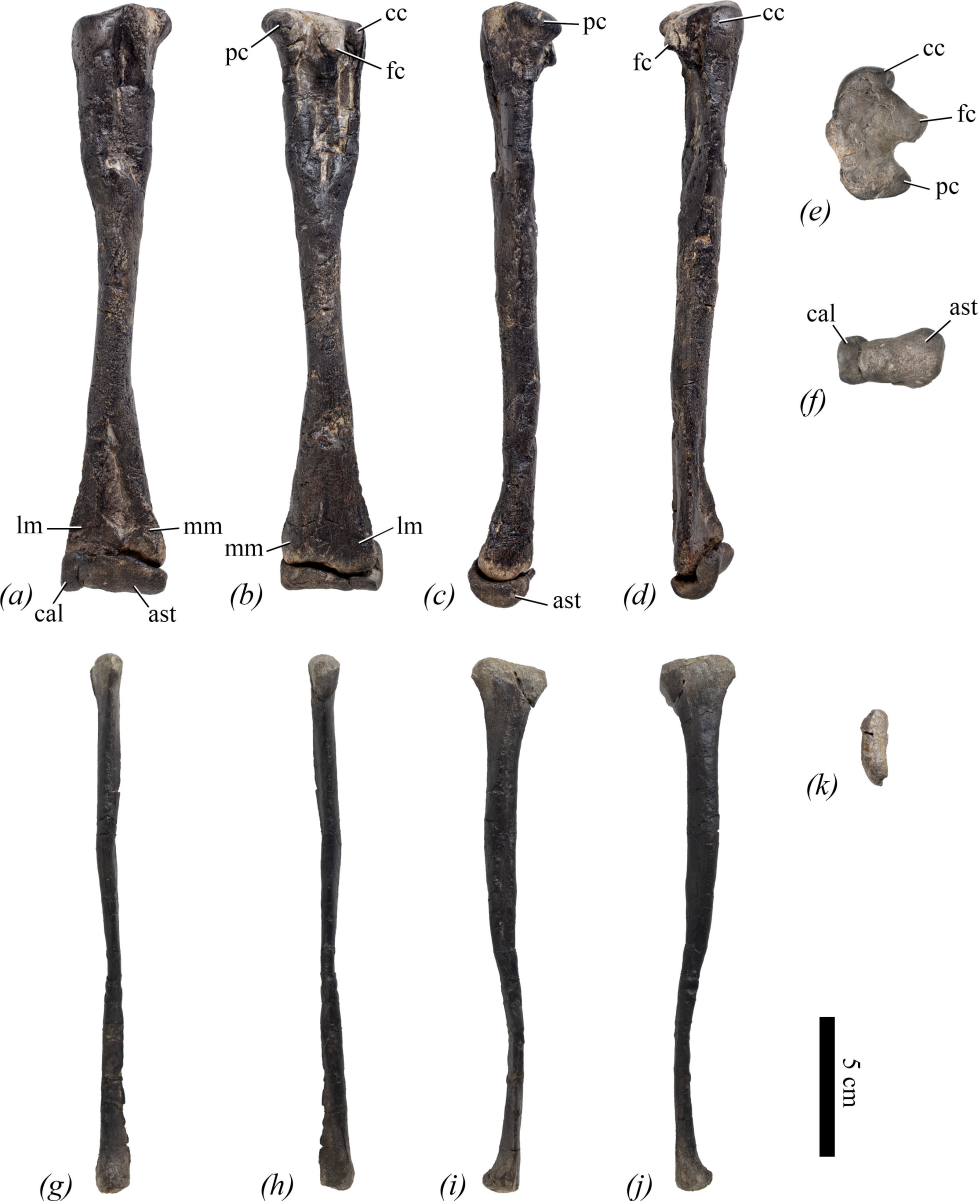
Right tibia (*a–e*), right astragalus (*a–d*,*f*) and left fibula (*a–k*) of NHMUK PV R 39000, *Enigmacursor mollyborthwickae*, in (*a,g*) anterior; (*b,h*) posterior; (*c,i*) medial; (*d,j*) lateral; (*e,k*) proximal; and (*f*) distal views. ast, astragalus; cal, calcaneum; cc, cnemial crest; fc, fibula condyle; lm, lateral malleolus; mm, medial malleolus; pc, posterior condyle. Scale bar, 5 cm.

The right tibia is complete (figure 12*a*–*e*) although the proximal end has been strongly crushed and rotated through 90° with respect to the distal end so that the fibula condyle projects posteriorly rather than laterally. The distal end of the left tibia is preserved but has also been crushed anteroposteriorly. The description therefore focuses on the more complete right tibia. Measurements are provided in table 2.

The following description is as preserved, rather than in the original anatomical orientation. In anterior or posterior view, the tibia consists of an elongated narrow shaft that connects the proximal and distal expansions. The proximal end of the tibia is expanded asymmetrically with respect to the shaft, with most of this expansion occurring medially (= posteriorly originally; figure 12*a*,*b*). The proximal end bears three distinct processes: a laterally (= anteriorly) projecting cnemial crest (figure 12*b*,*d*,*e*, cc); a posteriorly (= laterally) projecting fibular condyle (figure 12*a*,*d*,*e*, fc); and a posteromedially (= posteriorly) projecting posterior condyle (figure 12*b*,*c*,*e*, pc). The lateral margin of the cnemial crest is bluntly rounded and as preserved it is a mediolaterally narrow anteroposteriorly short, rounded process that is substantially smaller than either the fibular or posterior condyles. It is separated from the fibular condyle by a deep sulcus, but this has been accentuated by crushing. The fibular condyle has a sub-triangular outline in proximal view that tapers posteriorly to form a blunt rounded apex. An accessory condyle that is present on the anterior surface of the fibular condyle in *Eocursor parvus* [26], *Haya griva* [30], *Orodromeus makelai* [36] and *Hypsilophodon foxii* [31] is absent in *Enigmacursor*, *Lesothosaurus diagnosticus* [25], *Hexinlusaurus multidens* [27] and *Jeholosaurus shangyuanensis* [29]. The fibular condyle is separated from the posterior condyle by a second deep sulcus. The posterior condyle terminates in a blunt sub-triangular tip which forms a hook-like, medially directed process. This medially directed and hook-like posterior condyle is unusual and is absent in *Lesothosaurus diagnosticus* [25], *Eocursor parvus* [26], *Hexinlusaurus multidens, Yandusaurus hongheensis* [27], *Haya griva* [30], *Jeholosaurus shangyuanensis* [29] and *Hypsilophodon foxii* [31], but a similar feature appears to be present in *Thescelosaurus assiniboiensis* [53]. The proximal surface of the tibia is gently convex both anteroposteriorly and mediolaterally, and the entire surface is canted to face dorsally and slightly posteriorly (= laterally). The anterior margin of the cnemial crest forms a smooth continuous curve with the anterior (= medial) margin of the tibia, whereas the anteromedial (= posteromedial) corner forms a distinct angle.

In posterior view (figure 12*b*), the fibular condyle is situated slightly ventral to both the cnemial crest and posterior condyle. The cnemial crest appears to extend ventrally for approximately one-third of the length of the tibia, but due to crushing of the proximal end, it is not clear if this feature is genuine or if this simply represents plastic deformation of the proximal end of the tibia as it merges into the shaft. Ventral to the crushed proximal region, the shaft has a sub-triangular transverse cross section, with the apex of the triangle forming an intermuscular line that extends along the anterior midline of the shaft, whereas the posterior surface of the shaft is mediolaterally convex.

The distal end of the tibia has a sub-triangular outline in posterior view (figure 12*b*). The posterior surface of the distal end bears a shallow excavation but it is not clear if this is genuine or due to crushing. The distal end is subdivided into medial and lateral malleoli. The medial malleolus terminates slightly dorsal to the lateral malleolus and is separated from the latter by a distinct break in slope that forms a shallow sulcus between them for the reception of the astragalus. The medial malleolus is anteroposteriorly expanded with respect to the lateral malleolus so that the distal end of the tibia has a sub-triangular cross section that tapers laterally. In anterior view, the distal end bears a second large sulcus, although this appears to be the result of crushing. The anterolateral surface is flattened, presumably for articulation with the fibula.

The tibia is very similar in overall morphology to those of other small-bodied ornithischians such as *Lesothosaurus diagnosticus* [25], *Haya griva* [30], *Jeholosaurus shangyuanensis* [29] and *Hypsilophodon foxii* [31].

### 4.19. Fibula

The left fibula is complete (figure 12*g*–*k*), while only the distal half is preserved on the right. In lateral view (figure 12*i*), the left fibula is narrow and elongated with an anteroposteriorly expanded proximal end and a smaller posterior expansion distally. The proximal expansion is asymmetrical with respect to the shaft, extending slightly further posteriorly than it does anteriorly. The dorsal margin of the fibula is straight and merges with the posterior margin of the shaft around a smooth continuous curve forming a semicircular process in lateral view. By contrast, the dorsal surface of the fibula is separated from its anterior margin by a distinct break in slope and a more abrupt transition to the anterior margin of the shaft. In proximal view (figure 12*k*), the articular surface is mediolaterally narrow and anteroposteriorly elongated. Its medial margin is subtly concave while its anterior, lateral and posterior margins are convex, giving it a reniform outline.

Ventral to the proximal expansion, the shaft narrows in all dimensions. In medial view, a short but distinct ridge arises at a short distance below the proximal end and extends ventrally for a short distance before merging into the surface of the shaft. The shaft tapers in width ventrally. The proximal part of the shaft has a sub-elliptical transverse cross section whose long axis is oriented anteroposteriorly. At a point approximately halfway from the proximal end, the shaft is slightly kinked anteriorly and ventral to this, the cross section changes so that its long axis extends mediolaterally. The distal expansion is substantially smaller than the proximal expansion and has a sub-triangular outline. The anterior surface of the distal expansion is flat whereas its posterior surface is gently concave. In distal view, it has a sub-ovate outline with the long axis trending anteroposteriorly. The fibula is broadly similar in morphology to those of most other small-bodied ornithischians, including *Haya griva* [30], *Jeholosaurus shangyuanensis* [29], *Parksosaurus warreni* [52], *Hypsilophodon foxii* [31] and *Dryosaurus* sp. (CM 11340 [50]).

### 4.20. Tarsus

Of the tarsus, only the right astragalus is preserved, and it is mounted in association with the distal end of the right tibia and a reconstructed calcaneum, so its dorsal and lateral surfaces cannot be observed (figure 12*a*–*d,f*, ast). In distal view (figure 12*f*, ast), the astragalus is rectangular in outline with the long axis transverse and smoothly rounded corners. Its distal surface is strongly convex anteroposteriorly. In anterior and posterior views, the dorsal margin is essentially horizontal, lacking ascending processes on either side, although it is possible that these margins were broken and have been skimmed with plaster during reconstruction. The astragalus of *Lesothosaurus diagnosticus* [25], *Hexinlusaurus multidens* [27], *Jeholosaurus shangyuanensis* [29] and *Hypsilophodon foxii* [31] possess a well-defined anterior ascending process, but this feature is far less prominent in *Parksosaurus warreni* [52]. In *Enigmacursor*, the anterior margin is more dorsally elevated than the posterior margin. The posterior margin is slightly deflected ventrolaterally towards the reconstructed calcaneum. The medial surface of the astragalus is flat, and separated from the ventral, anterior and posterior surfaces by a gently rounded break in slope.

### 4.21. Pes

**Figure 13.**
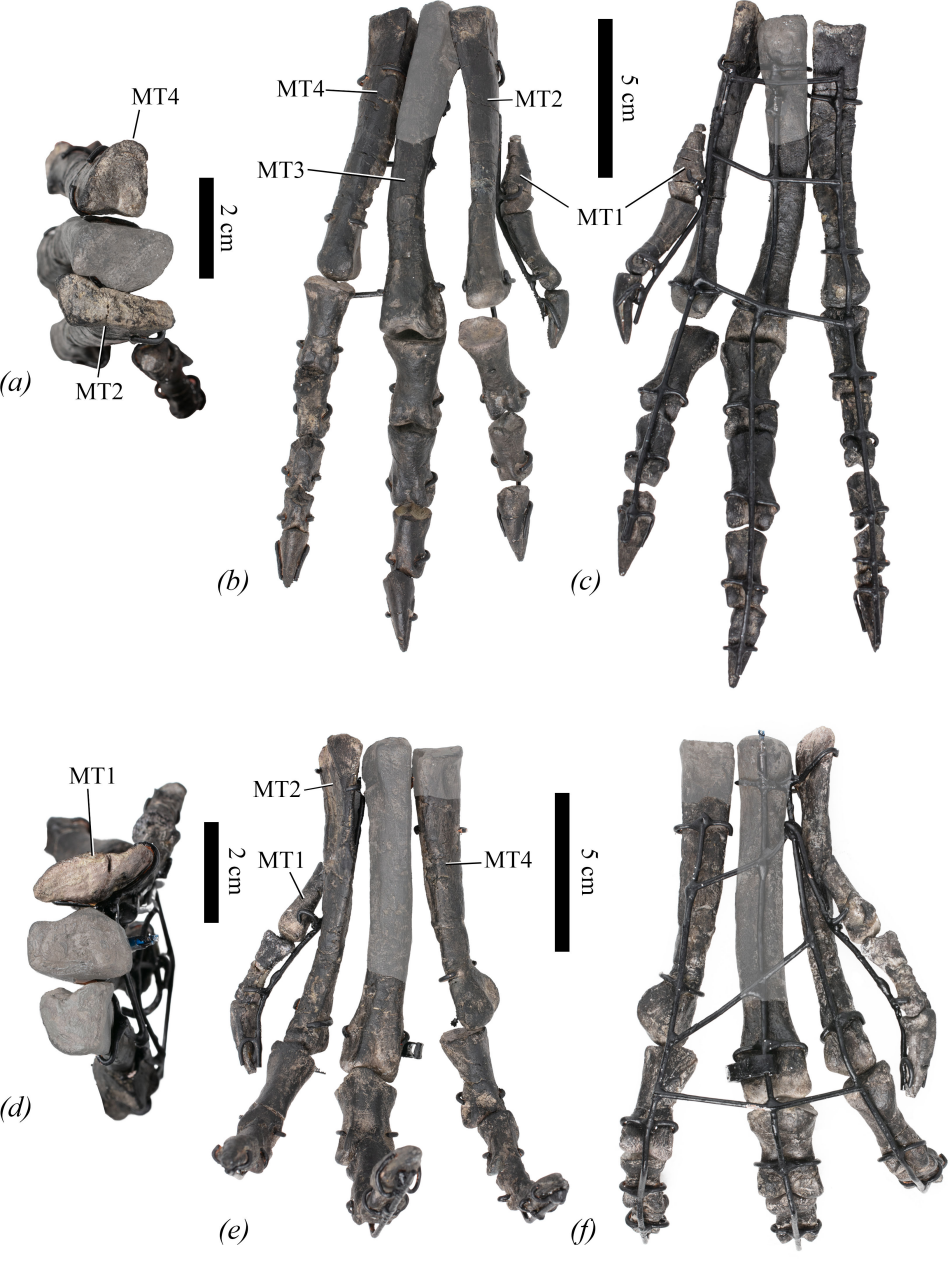
Pedes of NHMUK PV R 39000, *Enigmacursor mollyborthwickae*, in (*a–c*) right pes; (*d–f*) left pes in (*a*); (*d*) proximal; (*b,e*) anterior/flexor; and (*c,f*) posterior/plantar views. MT, metatarsal. The greyed out area indicates reconstruction. Scale bars, (*a*,*d*) 2 cm and (*b*,*c*,*e*,*f*) 5 cm.

The pes is almost complete on both sides. In the right pes (figure 13*a*–*c*) only the proximal ends of metatarsals (MT) 1 and 3 are missing, while on the left (figure 13*d*–*f*) the proximal ends of MT1 and 4 and most of MT3 (except its distal end) are not preserved. There is no evidence for a vestigial MT5 in either pes. Measurements are provided in table 2.

The pes is composed of four metatarsals, all of which bore phalanges. MT1 is short and appears to have been slender, although its proximal end is missing on both sides (figure 13, MT1). It is approximately half the length of the other metatarsals, has a roughly circular, slender shaft and is expanded both mediolaterally and anteroposteriorly at its distal end. In ventral view, it is roughly square in outline, with a gently concave posterior and lateral surface and flat anterior and medial surfaces. A raised lip extends around the medial and anterior articular surface, which is convex. MT1 is very similar to those of *Lesothosaurus diagnosticus* [25], *Hexinlusaurus multidens* [27], *Hypsilophodon foxii* (NHMUK PV R 196 [31]), *Parksosaurus warreni* [52] and most other small bipedal ornithischians, but differs from iguanodontians, such as *Dryosaurus* [50], in which MT1 is very reduced, and lacks phalanges.

MT2 is deeper anteroposteriorly than it is transversely, especially at its proximal end (figure 13*a*,*d*). In proximal view, it is rectangular, with the long axis oriented anteroposteriorly, and it is slightly wider anteriorly than posteriorly. The lateral surface of the proximal end and the entire shaft is flat, indicating close appression with MT3. The medial surface of the proximal end is also flat, but extending ventrally, the shaft becomes anteroposteriorly convex on its lateral surface, and the anterior, lateral and posterior surfaces merge into one another without breaks in slope. In contrast, the lateral surface of the shaft is divided from the anterior and posterior surfaces by strong ridges that extend the length of the metatarsal. The distal end of the MT2 is expanded mediolaterally but more so posteriorly; in distal view, it is square in outline with a concave posterior margin. This concavity separates the distal articular surface into two posterior condyles. The distal articular surface is convex, and clear ligament pits are lacking medially and laterally, although there are shallow concavities in this area.

As reconstructed, MT3 is the longest of the pes (figure 13, MT3). Its shaft is square in cross section, with flat lateral and medial surfaces indicating that the metatarsals would have been closely appressed in life. The medial and lateral surfaces are separated from the anterior and posterior surfaces by poorly defined ridges. The distal end of MT3 is expanded mediolaterally and a shallow groove separates the medial and lateral articular condyles in anterior view. In distal view, the metatarsal is rectangular in outline with a concave posterior surface, and the distal articular surface is saddle-shaped, being convex anteroposteriorly and concave mediolaterally. Shallow concavities are situated on the lateral and medial surfaces of the distal end but, as in MT2, these are not developed into deep ligament pits.

MT4 is shorter than MT2, but proximally it is mediolaterally broader (figure 13*a*,*d*). The proximal end is triangular in proximal view, with the apex pointing laterally. The medial surface of the proximal end is gently concave and separated from the anterior and posterior surfaces by distinct ridges. The anterior and lateral surfaces merge into each other without distinct breaks in slope, while the posterior surface is flat and separated from the lateral surface by a ridge that extends almost to the distal end of the metatarsal. Extending ventrally, the shaft is slightly laterally deflected at its ventral end, and ventral to this deflection, the medial surface is no longer flattened, but becomes convex, suggesting that MT4 was not as closely appressed, at least distally, as MT2 and MT3. MT4 is expanded posteriorly at its distal end, which is roughly square in distal view. It has gently concave medial and lateral surfaces, while the anterior and posterior surfaces of the distal end are gently convex. A distinct lip separates the articular surface from the anterior surface of the shaft. In distal view, the articular surface is convex.

The metatarsals are very similar to those of other small-bodied ornithischians, such as *Lesothosaurus diagnosticus* [25], *Hexinlusaurus multidens* [27], *Hypsilophodon foxii* [31], *Parksosaurus warreni* [52] and *Jeholosaurus shangyuanensis* [29]. However, in *Dryosaurus elderae* (CM 21 786) MT3 and MT4 differ in that proximally, MT3 possesses a notch on its lateral surface into which a medially directed process extending from MT4 articulates.

The phalangeal formula of *Enigmacursor* is 2-3-4-5-0, as in other small-bodied ornithischians, such as *Lesothosaurus diagnosticus* [25], *Hexinlusaurus multidens* [27], *Hypsilophodon foxii* [31], *Parksosaurus warreni* [52] and *Jeholosaurus shangyuanensis* [29]. All phalanges (Ph) are proximodistally longer than they are transversely wide. Ph 3-I is the largest; Ph 1-I, 2-I and 4-I are sub-equal to Ph 3-I in length but are significantly more slender, and Ph 1-I is the most slender of those of the first row. The phalanges become shorter and smaller from proximal to distal but are essentially similar in morphology. All are expanded at their proximal and distal ends both anteroposteriorly and mediolaterally, have concave proximal surfaces and convex distal surfaces, and have strongly developed collateral ligament pits on the medial and lateral surfaces of their distal ends. The ungual phalanges are all claw-shaped, much longer anteroposteriorly than the transverse width of their proximal ends, and bear lateral and medial grooves. The phalanges are identical in morphology to those of other small-bodied ornithischians.

## 5. Phylogenetic results

**Figure 14.**
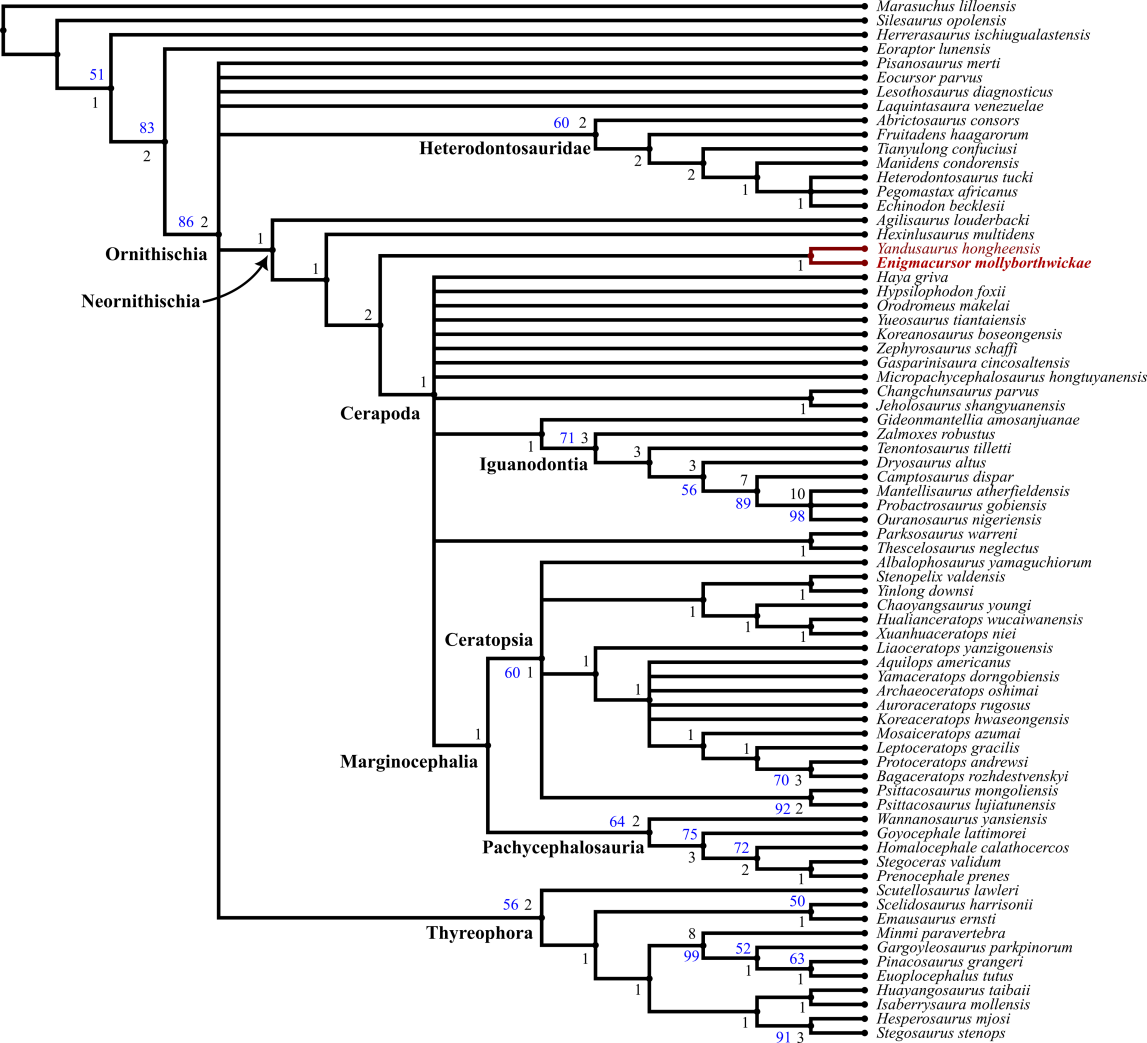
Strict consensus of 10 008 MPTs of length 1213 steps (consistency index = 0.369; retention index = 0.713). *Enigmacursor mollyborthwickae* and its sister taxon *Yandusaurus hongheensis* are highlighted in red. Numbers close to clades in black are decay indices (Bremer supports); those in blue are bootstrap values over 50%.

Analysis of the character–taxon matrix resulted in 10 008 MPTs with lengths of 1213 steps, a consistency index of 0.369 and a retention index of 0.713. A strict consensus of these (figure 14) found *Enigmacursor* to be the sister taxon to *Yandusaurus hongheensis*, and *Agilisaurus, Hexinlusaurus* and *Enigmacursor+Yandusaurus* as successive sister taxa to a poorly resolved cerapodan clade. This result is virtually identical to that obtained originally by Han *et al*. [17], except that the inclusion of *Enigmacursor* appears to have stabilized the phylogenetic position of *Yandusaurus*, which was originally part of a large basal cerapodan polytomy.

The sister–taxon relationship between *Yandusaurus* and *Enigmacursor* is supported by a single unambiguous synapomorphy, local to early diverging neornithischians: the humerus is strongly bowed medially in anterior view (character (char) 280). Characters that exclude *Enigmacursor+Yandusaurus* from Cerapoda include the absence of a diastema between the maxillary and premaxillary teeth (char 30; unknown in *Enigmacursor*), the possession of 15−20 maxillary teeth (char 203; unknown in *Enigmacursor*), a ventrally projecting ischiadic peduncle of the ischium (char 324), an absent or very weak femoral ligament sulcus (char 351) and an anterior trochanter that projects below the level of the femoral head and is separated from the greater trochanter by a notch in medial view (char 354). Characters that unite *Yandosaurus+Enigmacursor* with Cerapoda to the exclusion of *Hexinlusaurus* include ridges on cheek teeth confluent with marginal denticles (char 208; unknown in *Enigmacursor*), asymmetrical enamel on the crowns of cheek teeth (char 226; unknown in *Enigmacursor*), an angle of less than 150° between the pre- and postpubic processes (char 346), a trochanteric fossa separating the head from the greater trochanter (char 352) and an anterior trochanter with a reduced anteroposterior width and closely appressed to the greater trochanter (char 353). The relationship between *Yandusaurus* and *Enigmacursor* is weakly supported, with a bootstrap value of <50% and a decay index of 1; however, weak support is characteristic of most clades across the tree, except for those that are well established and not the focus of this phylogenetic analysis (e.g. Thyreophora, Ceratopsia, Heterodontosauridae).

## 6. Discussion

### 6.1. Phylogenetic implications

The results of the phylogenetic analysis are virtually identical to those reported originally by Han *et al*. [17], with a large basal polytomy of cerapodans, and no further resolution to the question of whether these small-bodied, bipedal taxa are stem-ornithopods, stem-marginocephalians, or lie basal to the split between the two (for a discussion of this problem, see [55]). However, the inclusion of *Enigmacursor* does stabilize the position of *Yandusaurus*, a poorly known taxon [27] that has previously behaved as a wildcard in ornithischian phylogenetic analyses [18]. To resolve the ‘basal cerapodan problem’, it is likely that we will need more complete skeletons of existing taxa, new taxa that could help to shed light on the order of character acquisition, detailed re-analyses of existing taxa in the light of new data, and new characters. Existing datasets are heavily focused on certain parts of the body, especially the skull, dentition and pelvis, and the axial skeleton is severely underrepresented (for example in [17], only 6/380 characters pertain to the postaxial cervicals and 5/380 to the dorsals). There is certainly more variation in the axial skeleton than currently represented in character lists, and new character discovery may help to elucidate the phylogenetic positions of many of these small-bodied taxa.

### 6.2. Taxonomic identity of other small-bodied ornithischians from the Morrison Formation

Seven taxa of small-bodied, non-iguanodontian ornithischians have been named from the Morrison Formation, many based on highly fragmentary material. These include the heterodontosaurid *Fruitadens haagarorum* [56], the early diverging ornithischians *Nanosaurus agilis* [5], *Nanosaurus* (= *Othnielia*) *rex* [6], *Laosaurus celer* [7], *Laosaurus gracilis* [7], *Laosaurus* (= *Othnielosaurus*) *consors* [8] and *Drinker nisti* [10]. The taxonomy of these animals has been revised periodically (e.g. [4,12,13]) and new material has been referred to some of them (e.g. [4,11,14]). Following examination of the historical type specimens, Barrett & Maidment [3] concluded that all previously named taxa except *Fruitadens* were *nomina dubia*, and that the material on which they were based represented indeterminate ornithischian or neornithischian remains or were juvenile individuals of *Dryosaurus*, and that *Drinker* could be an indeterminate pachycephalosaur.

*Enigmacursor* is represented by much more complete, three-dimensionally preserved skeletal remains than any previously named early diverging ornithischian specimen from the Morrison Formation, and it can be characterized by one possible autapomorphy and a unique character combination, primarily based on the femur (figure 1). This allows comparison with other partial skeletons and isolated femora from the Morrison Formation. The holotype of ‘*Nanosaurus agilis’* (YPM VP 001913) consists largely of natural moulds of a partial skeleton that includes impressions of two femora, but neither are well enough preserved to determine any morphological features [3], so they cannot be compared to the femora of *Enigmacursor*. Barrett & Maidment [3] concluded that YPM VP 001913 was an indeterminate neornithischian. A similarly preserved partial skeleton (DMNH 21716) described by Brill & Carpenter [11] likewise lacks morphological features of the femora that would allow comparison with *Enigmacursor* and, based on the description provided, could pertain to virtually any juvenile ornithischian. A very complete, articulated skeleton (NMZ 1000010, formerly SMA 0010) nicknamed ‘Barbara’ has not yet been described, but preliminary investigation by one of us (S.M.) suggests that the bone surfaces are poorly preserved and very crushed, precluding certain anatomical characteristics from being observed. However, as preserved, the fourth trochanter is crest-like rather than pendant. This could indicate that the specimen is referrable to *Enigmacursor*, but a closer examination is required to determine whether the trochanter is broken, and whether other features of its dorsal vertebrae, ilia, femora and tibiae are shared with *Enigmacursor*.

A partial skeleton, part of YPM VP 001882, which Marsh [8] proposed as the holotype of ‘*Laosaurus consors’* [3], preserves dorsal centra but these lack the autapomorphic offset of the anterior and posterior articular facets observed in *Enigmacursor*. The femur of YPM VP 001882 has an anterior trochanter that projects nearly to the same level as the greater trochanter, and a pendant fourth trochanter, different from the condition in *Enigmacursor*. BYU ESM 163R is a partial skeleton described by Galton & Jensen [14]. Like YPM VP 001882, this specimen lacks the offset anterior and posterior articular facets of the dorsals and possesses a femur in which the anterior trochanter closely approaches the greater trochanter in dorsal height and a pendant fourth trochanter [14], differing from *Enigmacursor*. Isolated femora are also known. The holotype of *‘Nanosaurus rex’* (YPM VP 001915) is an isolated, poorly preserved left femur. The anterior trochanter is broken dorsally, so the level of its dorsal projection cannot be determined, and the fourth trochanter is broken, so it is unknown if it was pendant, but the posterior surface of the head preserves a deep and well-defined ligament sulcus. Three femora from Quarry 9 at Como Bluff, USNM V 8397 and USNM 5808, are similar to YPM VP 001915, as far as can be determined, and all possess a similar ligament sulcus. The presence of this ligament sulcus distinguishes these specimens from *Enigmacursor*.

The Morrison Formation iguanodontian *Dryosaurus* possesses femora with an anterior trochanter that projects almost to the same level as the greater trochanter, a pendant fourth trochanter, and a prominently developed ligament sulcus on the posterior surface of the head [50], but YPM VP 001882, BYU ESM 163R, YPM VP 001915, USNM V 8397 and USNM 5808 differ from *Dryosaurus* in a number of respects. The scapulae of YPM VP 001882 and BYU ESM 163R both lack a supraglenoid fossa, a feature present in *Dryosaurus* (CM 11340) and many other early diverging iguanodontians [47]. The ilium of YPM VP 001882 lacks the very wide brevis shelf observed in *Dryosaurus* (CM 3392), and the femur of BYU ESM 163R lacks an anterior intercondylar groove. The presence of an anterior intercondylar groove is generally considered to be an iguanodontian synapomorphy [28], although Galton [50] stated that this feature was variably present in *Dryosaurus*. The isolated femora YPM VP 001915, USNM V 8397 and USNM 5808 also all lack an anterior intercondylar groove. YPM VP 001882, BYU ESM 163R, YPM VP 001915, USNM V 8397 and USNM 5808 are also not referrable to the small-bodied Morrison Formation heterodontosaurid *Fruitadens haagarorum*, because all these specimens possess a trochanteric fossa separating the head and greater trochanter, which is absent in the latter [57].

The aforementioned specimens are clearly not referrable to *Enigmacursor*, *Dryosaurus* or *Fruitadens* and this indicates that there was at least one other small-bodied neornithischian taxon present in the Morrison Formation fauna. USNM 5808 was referred to ‘*Drinker nisti’* by Bakker *et al*. [10], but because the diagnostic features of ‘*Drinker’* identified by Bakker *et al*. [10] were all dental, it is difficult to see how comparisons were made. All ‘*Drinker’* material currently resides in a private collection, and it has not been adequately described or illustrated [3,4], so it is not possible to make comparisons. The combination of characters observed in the femora of these specimens: a globose femoral head offset from the greater trochanter by a trochanteric fossa, an anterior trochanter approaching the height of the greater trochanter, and the lack of an anterior intercondylar groove, are features shared widely with other early diverging neornithischians, such as *Hypsilophodon foxii* [31], *Haya griva* [30] and *Jeholosaurus shangyuanensis* [29]. Consequently, the isolated femora YPM VP 001915, USNM V 8397 and USNM 5808 are indeterminate neornithischians. The mounted skeleton of YPM VP 001882 bears no unique features and is heavily reconstructed, and [3] concluded that it was also an indeterminate neornithischian. No autapomorphies or unique character combinations can be identified for BYU ESM 163R based on the description in Galton & Jensen [14], so it should currently be considered as indeterminate, but a redescription of the specimen is warranted in the light of more recent discoveries.

Galton [13] and Carpenter & Galton [4] mentioned ornithischian teeth and several dentaries (USNM V2771; USNM 5829; MWC 5822) that they considered to be early diverging neornithischians. Although the premaxillary teeth figured by Galton [13, fig. 3.10L,M] are similar in morphology to those of *Enigmacursor*, this morphology is shared with many other ornithischians (see Description) and cannot be used to refer them. The dentaries and cheek teeth do not overlap with elements preserved in *Enigmacursor*, and more complete skeletons will be required to determine whether they are referrable to this taxon.

### 6.3. Implications for ornithischian diversity in the Morrison Formation

Small ornithischians from the Morrison Formation have long been known but their diversity has been obscured by outdated taxonomic practices that failed to distinguish between symplesiomorphy and synapomorphy [3]. This led to a recent suggestion that all small-bodied ornithischians not referrable to *Fruitadens* belonged to a single taxon, ‘*Nanosaurus agilis’* [4]. Here, we demonstrate that there were at least two small-bodied bipedal neornithischians in the Morrison fauna: *Enigmacursor* and a second taxon, currently known only from indeterminate remains, but that was distinctly different from the former. It seems possible, however, that the diversity of these small dinosaurs might have been even greater. YPM VP 001915, ‘*Nanosaurus rex’*, an isolated femur with the morphology of the second taxon, is known from Felch Quarry 1 at Garden Park, near Cañon City, which is in the lower part of the Morrison Formation. Felch Quarry 1 is in the B3 systems tract of Maidment & Muxworthy [58], rocks that can be dated to the middle Kimmeridgian [3]. USNM V 8387 and USNM 5808, three more isolated femora with similar morphology, are known from Quarry 9 at Como Bluff (USNM collections data), which is high in the Morrison Formation, in the C6 systems tract of Maidment & Muxworthy [58] and dated to younger than 149.24 Ma (Tithonian [2]). This means that the USNM specimens were living at least 2 million years after YPM VP 001915, and perhaps considerably more. There remains the possibility, therefore, that these specimens do not represent the same taxon, and that diversity was even higher. More specimens are needed to test this hypothesis, but the need for detailed stratigraphic work accompanying the discovery and excavation of new specimens is of fundamental importance in disentangling true diversity.

## 7. Conclusions

NHMUK PV R 39000, a partial dinosaur skeleton from the Upper Jurassic Morrison Formation of Colorado, USA, represents a new genus and species of non-cerapodan neornithischian, which we name *Enigmacursor mollyborthwickae. Enigmacursor* is the most complete three-dimensionally preserved small ornithischian from the Morrison Formation and one of the most complete from the Middle–Late Jurassic globally. *Enigmacursor* can be distinguished from all other known specimens of Morrison Formation small ornithischians, demonstrating that many of these indeterminate remains likely represent a second, currently undiagnosable taxon. This indicates that although the fossil record of these small dinosaurs is poor, Morrison ornithischian species richness was likely higher than currently accepted.

## Data Availability

All data are included within this paper.
